# A method for intelligent allocation of diagnostic testing by leveraging data from commercial wearable devices: a case study on COVID-19

**DOI:** 10.1038/s41746-022-00672-z

**Published:** 2022-09-01

**Authors:** Md Mobashir Hasan Shandhi, Peter J. Cho, Ali R. Roghanizad, Karnika Singh, Will Wang, Oana M. Enache, Amanda Stern, Rami Sbahi, Bilge Tatar, Sean Fiscus, Qi Xuan Khoo, Yvonne Kuo, Xiao Lu, Joseph Hsieh, Alena Kalodzitsa, Amir Bahmani, Arash Alavi, Utsab Ray, Michael P. Snyder, Geoffrey S. Ginsburg, Dana K. Pasquale, Christopher W. Woods, Ryan J. Shaw, Jessilyn P. Dunn

**Affiliations:** 1 Department of Biomedical Engineering, Duke University, Durham, NC USA; 2 Department of Biostatistics & Bioinformatics, Duke University, Durham, NC USA; 3 Department of Genetics, Stanford University, Stanford, CA USA; 4 All of Us Research Program, National Institutes of Health, Bethesda, MD USA; 5 Department of Sociology, Duke University, Durham, NC USA; 6 Department of Population Health Sciences, School of Medicine, Duke University, Durham, NC USA; 7 Division of Infectious Diseases, Duke University Medical Center, Durham, NC USA; 8 Durham VA Medical Center, Durham, NC USA; 9 School of Nursing, Duke University, Durham, NC USA; 10 Duke Mobile App Gateway, Clinical and Translational Science Institute, Duke University, Durham, NC USA

**Keywords:** Predictive markers, Physiology

## Abstract

Mass surveillance testing can help control outbreaks of infectious diseases such as COVID-19. However, diagnostic test shortages are prevalent globally and continue to occur in the US with the onset of new COVID-19 variants and emerging diseases like monkeypox, demonstrating an unprecedented need for improving our current methods for mass surveillance testing. By targeting surveillance testing toward individuals who are most likely to be infected and, thus, increasing the testing positivity rate (i.e., percent positive in the surveillance group), fewer tests are needed to capture the same number of positive cases. Here, we developed an Intelligent Testing Allocation (ITA) method by leveraging data from the CovIdentify study (6765 participants) and the MyPHD study (8580 participants), including smartwatch data from 1265 individuals of whom 126 tested positive for COVID-19. Our rigorous model and parameter search uncovered the optimal time periods and aggregate metrics for monitoring continuous digital biomarkers to increase the positivity rate of COVID-19 diagnostic testing. We found that resting heart rate (RHR) features distinguished between COVID-19-positive and -negative cases earlier in the course of the infection than steps features, as early as 10 and 5 days prior to the diagnostic test, respectively. We also found that including steps features increased the area under the receiver operating characteristic curve (AUC-ROC) by 7–11% when compared with RHR features alone, while including RHR features improved the AUC of the ITA model’s precision-recall curve (AUC-PR) by 38–50% when compared with steps features alone. The best AUC-ROC (0.73 ± 0.14 and 0.77 on the cross-validated training set and independent test set, respectively) and AUC-PR (0.55 ± 0.21 and 0.24) were achieved by using data from a single device type (Fitbit) with high-resolution (minute-level) data. Finally, we show that ITA generates up to a 6.5-fold increase in the positivity rate in the cross-validated training set and up to a 4.5-fold increase in the positivity rate in the independent test set, including both symptomatic and asymptomatic (up to 27%) individuals. Our findings suggest that, if deployed on a large scale and without needing self-reported symptoms, the ITA method could improve the allocation of diagnostic testing resources and reduce the burden of test shortages.

## Introduction

The COVID-19 pandemic has severely impacted our world, with more than 562 million COVID-19 cases and 6.37 million deaths estimated worldwide^1^. In the US alone, there have been more than 90 million cases and 1 million deaths at the time of writing^2^. Mass surveillance testing has been identified as the most effective tool to monitor the spread of infectious diseases including COVID-19^3^. However, a combination of cost, availability, and impracticality of frequent and widespread testing impedes the mass epidemiologic surveillance needed to curb new disease outbreaks. To date, COVID-19 diagnostic test shortages are still prevalent globally, and shortages continue to occur in the US with the onset of new variants (e.g, Delta, Omicron)^4–6^. For example, when the Delta variant emerged in July 2021, daily demand for tests across the US surged from 250k to 1.5 million in the span of 2 months^7^. A similar circumstance occurred with the Omicron variant, where testing capacity failed to meet the sudden demand^8–10^. Inefficient diagnostic testing is also exacerbating the emerging threat of monkeypox in the US^11,12^. Furthermore, rural-urban disparities in testing access have worsened existing inequities resulting in further harm to underserved communities^13,14^. In June 2020, it was estimated that 64% of counties in the United States, predominantly rural, did not have access to COVID-19 testing^15^. Such circumstances lead to underreporting of COVID-19 incidence and may lead to a premature sense of security and unwarranted changes in public health measures^14^. Thus, there is an unprecedented need to improve our current and future methods for mass COVID-19 surveillance testing, especially as stronger testing capacity has been associated with reduced mortality and greater pandemic control^16^.

**Fig. 1:**
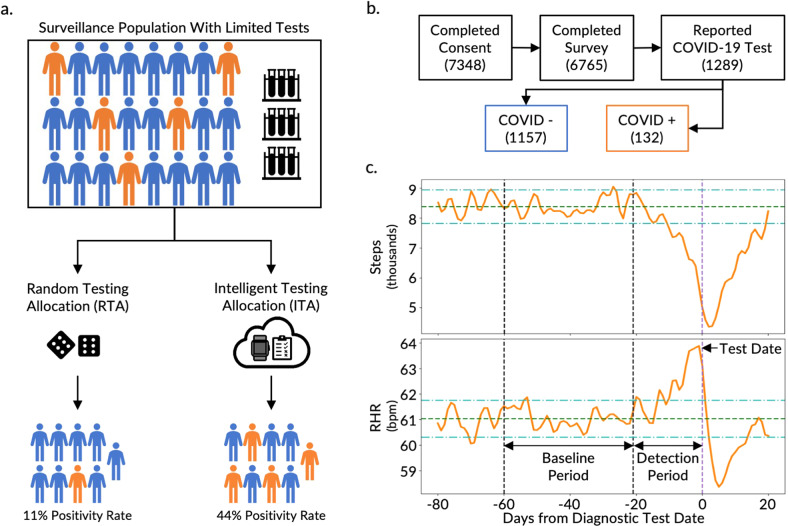
Overview of the Intelligent Testing Allocation (ITA) model, the CovIdentify cohort, and data. **a** Overview of the ITA model in comparison to a Random Testing Allocation (RTA) model that demonstrates the benefit of using the ITA model over existing RTA methods to improve the positivity rate of diagnostic testing in resource-limited settings. Human symbols with orange and blue colors represent individuals with and without COVID-19 infection, respectively. **b** A total of 7348 participants were recruited following informed consent in the CovIdentify study, out of whom 1289 participants reported COVID-19 diagnostic tests (1157 diagnosed as negative for COVID-19 and 132 diagnosed as positive for COVID-19). **c** The top panel shows the time-averaged step count and the bottom panel shows the time-averaged resting heart rate (RHR) of all participants (*n* = 50) in the training set (Supplementary Fig. 3, blue) who were tested positive for COVID-19 with the pre-defined baseline (between –60 and –22 days from the diagnostic test) and detection (between –21 and –1 days from the diagnostic test) periods marked with vertical black dashed lines. The dark green dashed lines and the light green dash-dotted lines display the baseline period mean and ± 2 standard deviations from the baseline mean, respectively. The light purple dashed vertical line shows the diagnostic test date.

By targeting surveillance testing toward individuals who are more likely to be infected with the disease, more positive cases can be captured with the same number of tests, increasing the positivity rate of the tested population (Fig. 1a)^4^. The positivity rate (i.e., percent positive rate or percent positive) is the percentage of all diagnostic tests performed that are positive. The likelihood of disease presence prior to a diagnostic test, or the pretest probability, is dependent on disease prevalence in the population under surveillance. By filtering the broader surveillance population to a subpopulation with a higher likelihood of infection, the allocation and utility of tests can be improved (Fig. 1a). In other words, more positive cases can be captured with the same number of tests and, thus, the testing positivity rate is increased. The development of tools to increase the testing positivity rate is not only crucial in the early phase of an infectious disease outbreak when the available clinical diagnostic testing tools are inadequate to meet the existing demand, but also throughout pandemics in remote locations, underserved communities, and low- and middle-income countries where testing is known to be particularly scarce^17^.

The rapid adoption of commercial wearable devices such as smartwatches and activity trackers brings forth opportunities to apply artificial intelligence methods toward the development of novel tools to support an intelligent disease detection infrastructure. Methods such as reinforcement learning or graph neural networks have already been proposed to aid contact tracing and surveillance testing^18,19^. Multiple studies suggest the utility of digital biomarkers, objective and quantifiable digitally collected physiological and behavioral data (e.g., resting heart rate (RHR), step count, sleep duration, and respiratory rate), collected by consumer devices along with patient-reported symptoms to monitor the progression of respiratory and influenza-like illnesses^20–27^. These studies emphasize the utility of wearables data as compared with symptom surveys or known exposure to COVID-19 as a result of its accessibility and scalability.

To determine who to test in settings where there are a limited number of diagnostic tests available (i.e., limited testing capacity), we explored whether information from wearables could help rank individuals by their likelihood of a current COVID-19 infection. To achieve this, we developed an Intelligent Testing Allocation (ITA) model that leverages differences in digital biomarkers to distinguish individuals who are likely positive or negative for COVID-19 in order to improve current methods of diagnostic test allocation and increase testing positivity rates.

## Results

We developed the CovIdentify platform in April 2020 to integrate commercial wearable device data and electronic symptom surveys to calculate an individual’s real-time risk of being infected with COVID-19. A total of 7348 participants e-consented to the CovIdentify study between April 2, 2020, and May 25, 2021, through the secure Research Electronic Data Capture (REDCap) system (Fig. 1b)^28^. Of those who consented, 6765 participants enrolled in the study (Supplementary Table 1) by completing an enrollment survey consisting of 37–61 questions that followed branching logic (Supplementary Note 1)^28^. Of those enrolled, 2887 participants connected their smartwatches to the CovIdentify platform, including 1689 Garmin, 1091 Fitbit, and 107 Apple smartwatches. Throughout the course of the study, 362,108 daily surveys were completed by 5859 unique participants, with a mean of 62 and a median of 37 daily surveys completed per individual. Of all CovIdentify participants, 1289 participants reported at least one diagnostic test result for COVID-19 (132 positive and 1157 negative) (Fig. 1b). All survey and device data collected through CovIdentify were transferred securely to a protected cloud environment for further analysis. Out of the 1289 participants with self-reported diagnostic test results, 136 participants (16 positive and 120 negative) had smartwatch data available during the time periods needed for analysis. These 136 participants had 151 ± 165 days of wearable data before the corresponding diagnostic test date. The relatively small number of participants with available smartwatch data out of the larger population is consistent with similar bring-your-own-device studies aimed at COVID-19 infection prediction from personal devices^22,23,27^.

### Development of the Intelligent Testing Allocation (ITA) model

A diagnostic testing decision support model was designed to leverage real-world data to intelligently allocate diagnostic tests in a surveillance population where there are insufficient tests available to test all people in the surveillance group (Fig. 1a, top). To increase the study population size, we augmented our dataset with data from the MyPHD study. Similar to CovIdentify, MyPHD collected simultaneous smartwatch, symptom, and diagnostic testing data during the COVID-19 pandemic^23,27^. The wearables and diagnostic testing data were publicly available^23,27^ while symptom data were added for this work. From the MyPHD study, smartwatch, symptom, and diagnostic testing data from an additional 1129 participants (110 positive and 1019 negative) were included in this analysis, including 53 ± 52 days of wearable data before corresponding diagnostic test dates.

### Differences in resting heart rate (RHR) and steps measured by smartwatches well before and immediately prior to a COVID-19 diagnostic test

To compare digital biomarkers between healthy and infected states, data were segmented into two time periods: a baseline period (22–60 days prior to the diagnostic test date) and a detection period (21 days prior to the diagnostic test date). We chose this window for the detection period to encompass the COVID-19 incubation period (2–14 days) reported by the CDC as well as the common delay between symptom onset and diagnostic testing. Consistent with prior literature^20,24^, daily RHR increased significantly during the detection period from baseline for those who were COVID-19 positive, with an average difference (±SD) of 1.65 ± 4.63 bpm (*n* = 117, *p* value <0.001, paired *t*-test) over the entire time periods. On average, daily RHR values more than two standard deviations from the baseline mean were present as early as 13 days prior to the positive test, with an increasing trend that peaked at 1 day prior to the test date (Fig. 1c, bottom). Conversely, the step count during the detection period decreased significantly from baseline, with a difference of –854 ± 2386 steps/day (*n* = 125, *p* value <0.0001, paired *t*-test). On average, step counts less than two standard deviations from the baseline mean were present as early as 10 days prior to the positive test and reached the minimum value 2 days after the test date (Fig. 1c, top). For the subset of participants in our dataset with available symptom onset dates, daily RHR and step count that differed beyond two standard deviations from the baseline mean occurred as early as 5 days before the symptom onset date (Supplementary Fig. 1). Timelines for this and other real-world infection studies should be considered as rough estimates because exact dates of exposure and symptom onset are unknown, unlike in controlled infection studies^26,29^. Our findings, however, are consistent with the 2–14-day COVID-19 incubation period reported by the CDC^30^.

There was also a significant difference in digital biomarkers between the baseline and detection periods of participants who tested negative, but it was less pronounced than for those who tested positive. Specifically, the daily RHR difference was 0.58 ± 4.78 bpm (*n* = 1094, *p* value <0.05, paired *t*-test) and the step count difference was –281 ± 2013 steps/day (*n* = 1136, *p* value <0.0001, paired *t*-test). We hypothesized that the digital biomarker differences in the COVID-19-negative group were because a subset of the negative group may have experienced a health anomaly other than COVID-19 (e.g., influenza) that resulted in physiological differences between the baseline and detection periods. Another recent study also observed RHR elevation and activity reduction in individuals who were COVID-19 negative but flu positive, and the magnitudes of these differences were lower than in individuals who were COVID-19 positive^22^. To explore the possibility that our COVID-19-negative group contains false negatives due to test inaccuracies or physiological differences due to a health anomaly besides COVID-19, we performed hierarchical clustering on the symptom data from individuals who reported negative tests and found a trend toward multiple subgroups (Supplementary Fig. 2). This finding supports the existence of COVID-19-negative subgroups. It should also be noted that the highly significant *p* value for the digital biomarker differences in the COVID-19-negative group is likely attributable to the higher number of participants (9-fold higher) compared with the COVID-19-positive group.

### Cohort definition

For the ITA model development, we only included subjects with sufficient wearable data (≥50% days with a device-specific minimum amount of data availability during periods of sleep for participants with high-frequency wearable data or ≥50% days with device-reported daily values for participants without high-frequency wearable data) in each of the baseline and detection periods. Sleep periods were defined as epochs of inactivity that occurred between midnight and 7 AM on a given day^27^. Consequently, 83 participants from CovIdentify (9 COVID-19 positive and 74 COVID-19 negative) and 437 participants from MyPHD (54 COVID-19 positive and 383 COVID-19 negative) were included in the ITA model development process (Table 1). Of the 63 COVID-19-positive cases, 24 had a clinically documented diagnosis, while the remainder were self-reported. Of the 520 participants with sufficient wearable data, 469 had high-frequency minute-level wearable data (280 from Fitbits) from which we calculated daily RHR and step counts. Device-reported daily values were available for the remaining 51 participants. To explore whether high-frequency wearable data or high-frequency wearable data from a single device type could improve the performance of digital biomarkers for ITA, we developed and validated our ITA model using three cohorts, which we refer to as (1) the All-Frequency (AF) cohort: participants with both high-frequency and device-reported daily values, (2) the All-High-Frequency (AHF) cohort: participants with high-frequency data only, and (3) the Fitbit-High-Frequency (FHF) cohort: participants with high-frequency Fitbit data only (Supplementary Fig. 3 and Supplementary Table 2). We analyzed these three cohorts separately in the subsequent analysis and compared the resulting ITA model performance. We divided each cohort into an 80% train and 20% test split, with FHF as a subset of AHF, which itself is a subset of AF to ensure that no observations in the training set of one cohort existed in the test set of another (Supplementary Fig. 3).

**Table 1:** Summary of the cohorts.

Cohort	Total *N* (Test *N*)	Total COVID+ (test)	Total COVID– (test)
All-Frequency (AF)	520 (105)	63 (13)	457 (92)
All-High-Frequency (AHF)	469 (97)	54 (11)	415 (86)
Fitbit-High-Frequency (FHF)	280 (63)	40 (7)	240 (56)

Total refers to training + test data.

To explore differences in digital biomarkers (median or mean) between the detection and baseline periods that may be useful for the development of ITA model features, we designed four deviation metrics including (1) Δ (detection – baseline), (2) normalized Δ, (3) standardized Δ, and (4) *Z*-score ((detection – baseline mean) / baseline standard deviation) (Table 2). Each of the four deviation metrics was calculated on the training data by digital biomarkers (RHR and step count), day in the detection period, and cohort (examples in Supplementary Figs. 4 and 5), resulting in four calculated metrics per cohort per biomarker. These training data deviation metrics were used as inputs into the subsequent statistical analysis for feature extraction and the ITA model training. We extracted the same resultant features from the independent test set for subsequent ITA model evaluation.

**Table 2:** Features extracted from the digital biomarkers (DBs) for the development of ITA algorithm.

Metric	Definition	Equation
*Deviation metrics*		
Delta (Δ)	Deviation in digital biomarker from baseline median value	DB_Detection_ – DB_Baseline, Median_
Delta_Normalized	Delta normalized by baseline median value	((DB_Detection_ – DB_Baseline, Median_) / DB_Baseline, Median_)
Delta_Standardized	Delta standardized by baseline median and interquartile range (IQR)	((DB_Detection_ – DB_Baseline, Median_) / DB_Baseline, IQR_)
*Z*-score	Deviation in digital biomarker from baseline mean, standardized by baseline standard deviation (SD)	((DB_Detection_ – DB_Baseline, Mean_) / DB_Baseline, SD_)
*Summary statistics (features)*		
Average	Average of interday deviation metrics	
Median	Median of interday deviation metrics	
Maximum	Maximum of interday deviation metrics	
Minimum	Minimum of interday deviation metrics	
Range	Range of interday deviation metrics	

**Fig. 2:**
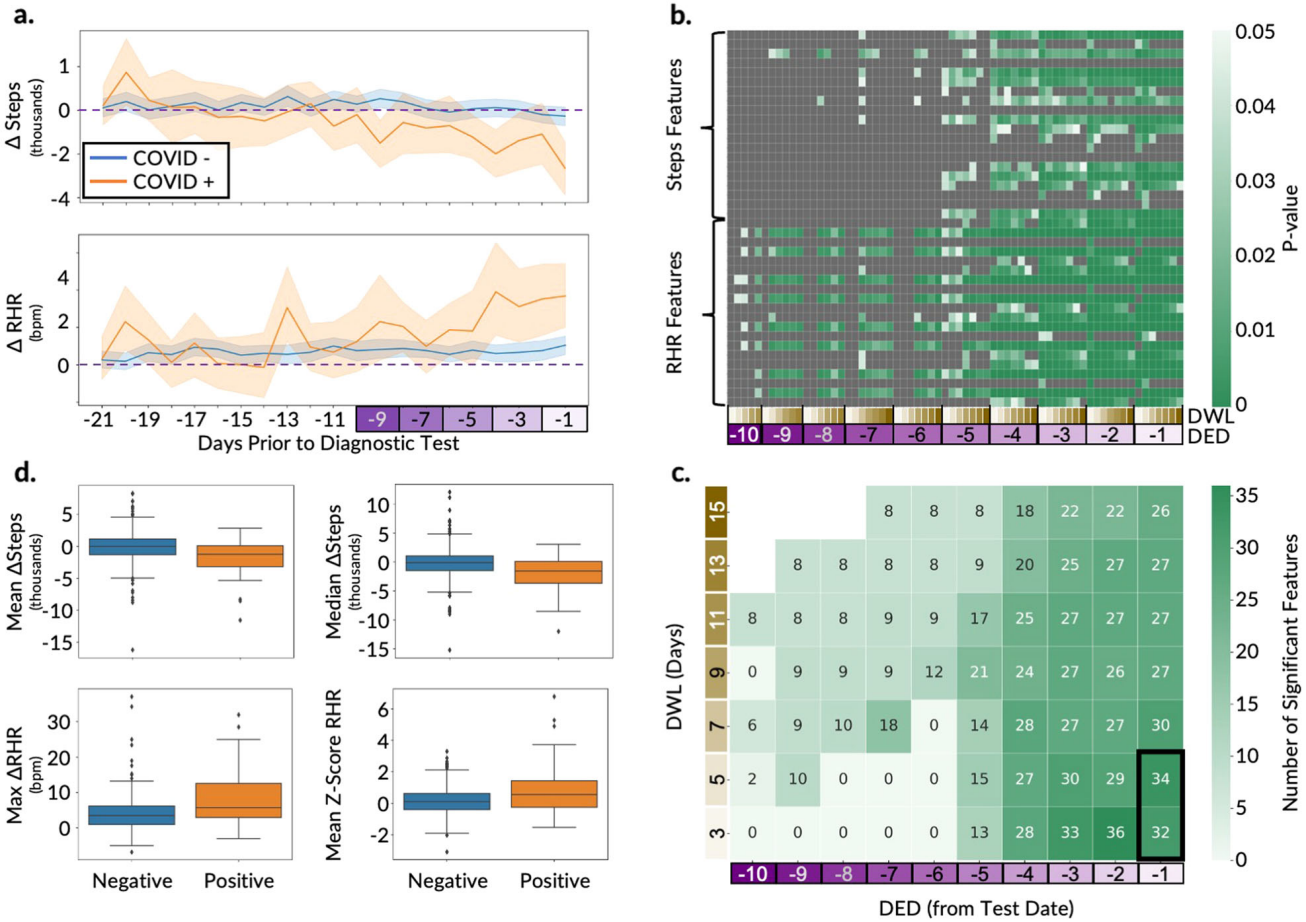
Overview of digital biomarker exploration and feature engineering for the ITA model development on the AF cohort. **a** Time-series plot of the deviation in digital biomarkers (ΔSteps and ΔRHR) in the detection window compared to baseline periods, between the participants diagnosed as COVID-19 positive and negative. The horizontal dashed line displays the baseline median and the confidence bounds show the 95% confidence intervals. **b** Heatmaps of steps and RHR features that are statistically significantly different (*p* value <0.05; unpaired *t*-tests) in a grid search with a different detection end date (DED) and detection window length (DWL) combinations, with green boxes showing *p* values <0.05 and gray boxes showing *p* values ≥0.05. The *p* values are adjusted with the Benjamini–Hochberg method for multiple hypothesis correction. **c** Summary of the significant features (*p* value <0.05; unpaired *t*-tests) from **b**, with each box showing the number of statistically significant features for the different combinations of DED and DWL. The intersection of the significant features across DWL of 3 and 5 days with a common DED of 1 day prior to the test date (as shown using the black rectangle) was used for the ITA model development. **d** Box plots comparing the distribution of the two most significant steps and RHR features between the participants diagnosed as COVID-19 positive and negative. The centerlines denote feature medians, bounds of boxes represent 25th and 75th percentiles, whiskers denote nonoutlier data range and the diamonds denote outlier values.

On average, step count decreased (ΔSteps) significantly from baseline to the detection period in COVID-19-positive versus -negative participants (574 vs. 179, 479 vs. 234, and 601 vs. 216 steps per day for the AF, AHF, and FHF training data, respectively; *p* value <0.05, unpaired *t*-tests) (Fig. 2a and Supplementary Figs. 6a and 7a, top plots). Conversely, RHR increased (ΔRHR) significantly from baseline to the detection period in COVID-19-positive versus -negative participants (1.8 vs. 0.7, 1.9 vs. 0.8, and 1.8 vs. 0.7 bpm for the AF, AHF, and FHF training data, respectively; *p* value <0.05, unpaired *t*-test) (Fig. 2a and Supplementary Figs. 6a and 7a, bottom plots). The 95% confidence intervals of the mean ΔSteps and the mean ΔRHR overlap considerably between positive and negative participants for the initial phase of the detection period (approximately 21–5 days prior to the test date). However, closer to the diagnostic test date (approximately 4–1 day prior to the test date) the 95% confidence intervals of mean ΔSteps largely do not overlap, and the 95% confidence intervals of mean ΔRHR do not overlap at all (Fig. 2a). The fact that the 95% confidence intervals of mean ΔSteps and mean ΔRHR do not overlap later in the detection period is consistent with prior literature^31^ and suggests that it is possible to aggregate data into summary statistics to develop a decision boundary that effectively separates COVID-19-positive and -negative cases. However, the overlap in estimated mean values prior to day 5 suggests that separation between positive and negative cases may be more challenging prior to that point in time. Although the 95% confidence intervals closer to the test date were non-overlapping, there was overlap in the variance of the digital biomarkers between the two groups during that time period (Supplementary Fig. 8), which may hinder model performance as separation of the 95% confidence intervals does not necessarily imply significant differences between the groups^32^. Similar estimates of variability have not been reported prior, so we were unable to compare our mean statistics variability to prior literature.

To maximize the separability of the COVID-19-positive and -negative groups in the training set, we performed statistical analysis to explore how different lengths and start times of the detection window, parametrized respectively by two variables (the detection end date, defined by days prior to the diagnostic test date, and the detection window length defined by number of days), would affect the separation between these two groups. We performed a combinatorial analysis across these two parameters (detection end date and detection window length) to calculate five summary statistics (mean, median, maximum, minimum, and range) of the four deviation metrics (Table 2) to be used as features for model building. This resulted in 40 total summary statistics (20 each from steps and RHR), which we refer to as steps and RHR features, respectively. Statistical comparison of the steps and RHR features between the COVID-19-positive and COVID-19-negative groups was performed on the training data for the AF, AHF, and FHF cohorts separately to uncover the statistically significant features (unpaired *t*-tests; Benjamini–Hochberg corrected *p* value <0.05).

A systematic grid search to optimize the detection end date and detection window length demonstrated that the closer the detection period is to the diagnostic test date, the larger the number of features that are significantly different between the COVID-19-positive and -negative groups (Fig. 2b and Supplementary Figs. 6b and 7b). Across all evaluated detection end dates, the day prior to the diagnostic test date (detection end date = –1) generated the largest number of significant features for all cohorts. Also, across all cohorts, there were more significant RHR features than steps features (Fig. 2b and Supplementary Figs. 6b and 7b). Additionally, RHR features became significant earlier in the detection period than steps features (detection end date as early as –10 vs. –5 days, respectively), which indicates that changes in RHR occur earlier than steps during the course of infection. Comparison across the three cohorts revealed AF generated the highest number of significant features compared with the AHF and FHF cohorts, which may be attributable to the larger population size of AF. This demonstrates the tradeoff in wearables studies between high-frequency data, which is less common but contains more information, and larger population data, which contains data at a variety of sampling frequencies but overall more data to train the models. Across detection window length values, 3 and 5 days generated the largest number of significant features for all cohorts (Fig. 2c and Supplementary Figs. 6c and 7c), while 5 days also corresponded to the date of the maximum divergence between ΔSteps and ΔRHR (Fig. 2a). Ultimately, this systematic analysis pointed to an optimal detection end date of 1 day prior to the diagnostic test date and an optimal detection window length of 5 days for the detection window duration, both of which were used to generate features for the ITA model.

When implementing the detection end date timepoint and detection window length duration that best separated the COVID-19-positive and -negative groups, there were 28–31 significant features (*p* value <0.05; unpaired *t*-tests with Benjamini–Hochberg multiple hypothesis correction) that overlapped across the three cohorts, indicating their robustness to differences in data resolution and device types (Supplementary Table 3). The top 7–9 features, ranked in order of significance, originated from the RHR digital biomarker. To gain a more mechanistic understanding of the RHR and step digital biomarkers, we explored the top two most significantly different (lowest *p* value) features for each digital biomarker between those who were COVID-19-positive or -negative in the AF cohort (Fig. 2d). The decrease in steps during the detection period as compared to baseline was greater in those with COVID-19, with a 2054 vs. 99 median decrease in steps (median ΔSteps) and a 1775 vs. 64 mean decrease in steps for those who were COVID-19 positive vs. those who were COVID-19 negative, respectively (*p* values <0.0001; unpaired *t*-tests with Benjamini–Hochberg multiple hypothesis correction). Conversely, the increase in maximum deviation in RHR in the detection period as compared to baseline (maximum ΔRHR) and the increase in mean of *Z*-scores in the detection period as compared to baseline (mean of *Z*-score RHR) were both significantly higher for COVID-19-positive participants compared to COVID-19-negative participants (8.4 vs. 4.3 bpm for maximum ΔRHR and 0.9 vs. 0.2 for the mean of *Z*-score-RHR; *p* values <0.0001; unpaired *t*-tests with Benjamini–Hochberg multiple hypothesis correction). Consistent across all three cohorts, the median and mean ΔSteps were the most significant (lowest *p* value) steps features (Supplementary Figs. 6d and 7d). However, the top two RHR features differed, which were median and mean *Z*-score-RHR, and maximum ΔRHR and maximum of normalized ΔRHR for the AHF and FHF cohorts, respectively (Supplementary Figs. 6d and 7d and Supplementary Table 3). The observation of the same top two steps features given the differences in the top two RHR features across the three cohorts may originate from the resolution and device-reported digital biomarkers. For example, the definition of a step and the calculation of the daily step count may be more similar across different device types, while the RHR definition and available HR data resolution may vary more substantially across device types. Although these top features are significantly different between those who are COVID-19 positive and negative, their distributions do overlap, even though the tailedness varies in direction and extent (Fig. 2d and Supplementary Figs. 6d, 7d, and 9), which points to broader challenges surrounding predictive modeling efforts using standard consumer wearable device data for COVID-19 infection detection.

To achieve our broader goal of determining who should receive a diagnostic test under circumstances where there are limited tests available, we aimed to design a model that outputs the probability of a person being infected. However, because our ground truth information is binary (positive or negative for COVID-19), we designed this model as a binary classifier that enabled a straightforward evaluation of its performance. We used the features that were significantly different in the training data between those who were COVID-19 positive and negative (29 features for AF, 28 for AHF, and 31 for FHF) as inputs into five machine learning classification models: logistic regression, k-nearest neighbors, support vector machine, random forest, and extreme gradient boosting (Supplementary Table 4). We chose these five well-established classification models to explore how increasing model complexity and the addition of non-linearity impact the model performance. We trained these classification models on the training data using nested cross-validation (CV) with an inner loop for hyperparameter tuning and an outer loop for model selection. We chose recall as our preferred scoring metric for model selection and evaluation to emphasize the relative impact/cost of false negatives compared to false positives, as an individual who is truly positive for COVID-19 and is wrongly classified as negative (or healthy) would further spread disease.

**Fig. 3:**
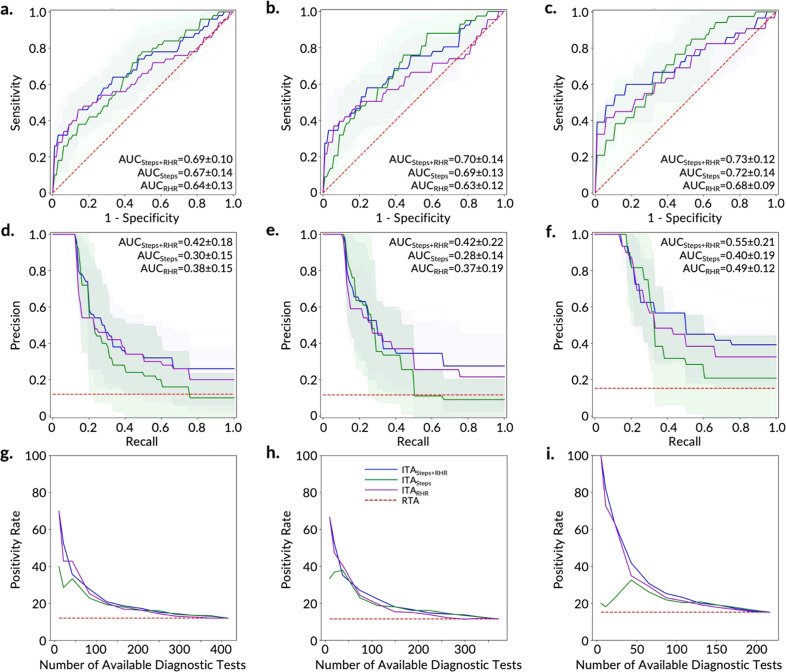
Prediction and ranking results of the ITA models on the training sets for the AF (a, d, and g), AHF (b, e, and h), and FHF (c, f, and i) cohorts using features from a combination of Steps and RHR (blue), Steps (green), and RHR (violet) digital biomarkers. **a**–**c** Receiver operating characteristics curves (ROCs) and **d**–**f** precision-recall curves (PRCs) for the discrimination between COVID-19-positive participants and -negative participants in the training set. The light blue, light green, and light violet areas show one standard deviation from the mean of the ROCs/PRCs generated from 10-fold nested cross-validation on the training set and the red dashed line shows the results based on a Random Testing Allocation (RTA) model (the null model). **g**–**i** The positivity rate of the diagnostic testing subpopulation as determined by ITA given a specific number of available diagnostic tests. The red dashed line displays the positivity rate/pretest probability of an RTA (null) model.

**Fig. 4:**
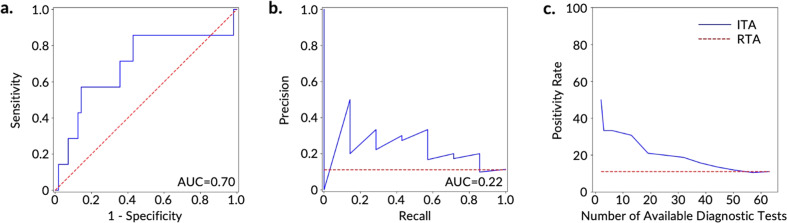
Prediction and ranking results of the ITA models on the test set of the FHF cohort using RHR digital biomarkers. **a** ROC and **b** PRC for the discrimination between COVID-19-positive participants (*n* = 7) and -negative participants (*n* = 56). The red dashed line shows the results based on an RTA model. **c** Positivity rate of the diagnostic testing subpopulation as determined by ITA given a specific number of available diagnostic tests. The red dashed line shows the positivity rate of an RTA (null) model.

Following training, we evaluated the performance of the trained model on the independent test set and used two well-established reporting metrics, including the most commonly reported metric for studies of this kind (the area under the curve for the receiver operating characteristic curve (AUC-ROC))^24,33–37^, and the metric that is most appropriate for this classification task (AUC for the precision-recall curve (AUC-PR))^38^ (Supplementary Table 3, Figs. 3 and 4, and Supplementary Fig. 10). AUC-PR is more appropriate with class-imbalanced data^38,39^, which is the case here (12–15% COVID-19 positive and 85–88% negative in each of the three cohorts). The class imbalance in our dataset was not correctable through resampling methods—we have observed that distributions of features overlap between the COVID-19-positive and -negative participants, as demonstrated in the individual feature comparison (Fig. 2d and Supplementary Figs. 6d and 7d), as well as in the low dimensional representation (using principal component analysis and t-stochastic neighbor embedding) of all the features in the training set of the AF cohort (Supplementary Fig. 11).

Of the five models tested, logistic regression outperformed all other models based on the training AUC-PR for all three cohorts and was also the best performing model based on the training AUC-ROC for the AF and FHF cohorts. The superior performance of the logistic regression among other (more complex and nonlinear) models may be attributed to the tendency of more complex and nonlinear models to overfit the training data^40^, which comes to light with our CV methods. The superior performance of the logistic regression also points to the potential to develop explainable machine learning predictive models for the ITA model that enables rapid translation from bench to bedside. Overall, the classifier performed best in the FHF cohort (Supplementary Table 3, Fig. 3c, f, and Supplementary Fig. 10c, f), followed by the AHF cohort, (Fig. 3b, e and Supplementary Fig. 10b, e) and finally the AF cohort (Fig. 3a, d and Supplementary Fig. 10a, d). These performance differences indicate that device-related and data resolution differences may confound disease-related physiological differences captured by digital biomarkers. Therefore, building models using a single device type and with higher resolution data improves performance. For the FHF cohort, the logistic regression model resulted in an AUC-ROC of 0.73 ± 0.12 and AUC-PR of 0.55 ± 0.21 on the cross-validated training set (Fig. 3c, f), and AUC-ROC of 0.77 and AUC-PR of 0.24 on the test set (Supplementary Fig. 10c, f). The AUC-ROC from the models were similar to those reported in recent similar studies^24,34,37^.

However, the performance of the models based only on AUC-ROC in the context of imbalanced data can be misleading, as a large change in the number of false positives may have a small effect on the false-positive rate^39^. The precision metric, which integrates both true positives and false positives, can mitigate the effect of an imbalanced dataset (e.g., the higher proportion of negatives seen in this type of data) on a model’s performance. Our precision-recall analysis (Fig. 3d–f and Supplementary Fig. 10d–f) demonstrates that we can improve the recall (minimizing false negatives) at the expense of precision. In an extreme example, we were able to achieve 100% recall with a precision of 0.4 on the cross-validated training set of the FHF cohort, whereas, a dummy classifier with random chance (i.e., Random Testing Allocation (RTA)) can achieve a precision of 0.15 on this dataset. It is also important to note that we are not considering resource-limited settings in the ROC and PR analysis; instead, it is assumed that there are a sufficient number of diagnostic tests available for the entire surveillance group. In a resource-limited setting, 100% recall may not be achievable due to the shortage of diagnostic testing.

To understand the relative contribution of the steps and RHR digital biomarkers to the ITA model performance, we developed two separate sets of models using features based only on either steps or RHR using the training set data with logistic regression, and later validated on the test set. Consistent with previous literature^24,34^ the models using steps-based features alone had a higher AUC-ROC than models using RHR-based features alone (cross-validated AUC-ROC of 0.67 vs. 0.64, 0.69 vs. 0.63, and 0.72 vs. 0.68 for steps vs. RHR features for the AF, AHF, and FHF training sets, respectively) (Fig. 3). Interestingly, when using the AUC-PR as the performance metric, models using features based on RHR digital biomarkers outperformed models using features based on steps digital biomarkers, a finding that has not been previously reported (cross-validated AUC-PR of 0.30 vs. 0.38, 0.28 vs. 0.37, and 0.40 vs. 0.49 for steps and RHR features for the AF, AHF, and FHF training datasets, respectively) (Fig. 3). The validation on the test sets also demonstrated similar results (AUC-ROC of 0.61 vs. 0.60, 0.66 vs. 0.58, and 0.71 vs. 0.70 and AUC-PR of 0.16 vs. 0.18, 0.17 vs. 0.17, and 0.18 vs. 0.22 for steps vs. RHR features for the AF, AHF, and FHF test sets, respectively) (Supplementary Fig. 10). Overall, the addition of steps features increased the AUC-ROC of the ITA model by 7–11% compared with RHR features alone, while RHR features improved the AUC-PR of the ITA model by 38–50% compared with steps features alone on the training set. In other words, the exclusion of each steps and RHR features individually decreased the AUC-ROC of the ITA model by 7–10% and 1–3% for the training set (5–11% and 2–9% for the test set), respectively, compared to the ITA model with both steps and RHR features (Fig. 3a–f and Supplementary Fig. 10a–f). On the other hand, the exclusion of each steps and RHR features individually decreased the AUC-PR of the ITA model by 10–12% and 19–27% for the training set (5–15% and 5–25% for the test set) compared to the ITA model with both steps and RHR features. These results suggest that, while steps features provide more salient information on the tradeoff between the true-positive rate and false-positive rate, RHR features provide more salient information on the tradeoff between the true-positive rate and the precision (positive predictive value). In other words, while steps features improved the specificity of the predictive model, RHR features improved the precision.

In addition to comparing the performance of ITA models with steps and RHR features alone to ITA models with both steps and RHR features on both training and test set, we also compared the relative feature importance in the logistic regression model using both steps and RHR features on the training set. Our results demonstrated that two, one, and four of the top five features originated from RHR in the AF, AHF, and FHF cohorts, respectively, with the remaining features originating from steps (Supplementary Fig. 12). In all three cohorts, median ΔSteps and mean ΔSteps were the two most important steps features, which was consistent with our earlier statistical analysis. Maximum ΔRHR was the most important RHR feature for the AF and AHF cohorts and the second most important RHR feature for the FHF cohort, and was also one of the top two most significant features in our earlier statistical analysis for the AF and FHF cohorts.

### Improvement in positivity rate for COVID-19 diagnostic testing using the ITA method

We next evaluated how the ITA model can improve the current standard of practice for COVID-19 infection surveillance. Under current surveillance testing methods in the US, while some tests are taken due to symptoms or possible exposure, many are taken as precautionary measures for traveling or for surveillance in schools and workplaces^30^. While such forms of widespread RTA surveillance are beneficial, the positivity rate of widespread diagnostic testing is typically low and, thus, requires sufficient testing capacity in order to prevent testing shortages (e.g., sold out at-home testing kits). Applying an equivalent RTA surveillance approach to our study population results in a 12% positivity rate in both our AF-training (50 COVID-19-positive participants out of 365 participants in total) and AF-test (13 COVID-19-positive participants out of 92 participants in total) datasets. It is important to note that the 12% positivity rate is consistent for all levels of diagnostic testing capacity (0–100% of population). When employing ITA with both steps and RHR features, and adding the constraint of limited diagnostic testing capacity (10–30% of population), the testing positivity rate of the cross-validated model increased 2–3 fold (21–36% positivity rate) for the training dataset (Fig. 3g) and 1.5–2.5 fold (19–29% positivity rate) for the testing dataset (Supplementary Fig. 10g).

A comparison of the three cohorts demonstrated that the best performing ITA model with both steps and RHR features stemmed from the FHF cohort and was followed by the AHF cohort (Fig. 3h, i and Supplementary Fig. 10h, i). By utilizing ITA and assuming a diagnostic testing capacity at 10–30% of the population, the positivity rate of the FHF and AHF cross-validated training datasets increased by 4 fold (64% positivity rate) and 3 fold (35% positivity rate) when compared to the RTA positivity rates of 15% and 12% for FHF and AHF cohorts, respectively. For the FHF cohort, the positivity rate further increased up to 6.5 fold (100% positivity rate) in the cross-validated training dataset when the diagnostic testing capacity was reduced to 2.5–5% of the population (5–11 diagnostic tests to be allocated to individuals in the training dataset) (Fig. 3i). Using the independent test dataset with both steps and RHR features, the positivity rate of the FHF and AHF cohorts increased by 1.5–3 fold (17–31% positivity rate) and 2–3 fold (21–32% positivity rate), respectively, compared to the RTA positivity rate of 11%, when the diagnostic testing capacity was 10–30% of the population. These results indicate the potential of the ITA model to target diagnostic testing resources toward individuals who have a higher likelihood of testing positive (i.e., increasing the positivity rate of diagnostic testing) and enable more efficient allocation of testing capacity. When we compared the ITA model performance in terms of improving the positivity rate of the diagnostic testing in a resource-limited setting among models with steps and RHR features separately and together, the results demonstrated that ITA models using only RHR features often achieved similar performance (similar positivity rate) on the training set and similar and in some cases even better performance (further improved positivity rate) on the test set in comparison with the models that used both steps and RHR features together (Fig. 3g–i and Supplementary Fig. 6g–i). For example, the ITA model using only RHR features improved the positivity rate up to 4.5 fold (positivity rate of 50%) compared to the RTA positivity rate of 11% on the test set of FHF cohort (Supplementary Fig. 10i). The superior performance of the ITA model using RHR-only features over the ITA model using steps-only and the ITA model using both steps and RHR features may be attributed to the nonspecific nature of the steps features, which can experience changes unrelated to COVID-19 (other diseases, quarantine, stress, etc.). These results demonstrate the potential to develop an ITA system to allocate diagnostic testing in limited resource settings only using physiological digital biomarkers without relying on potentially nonspecific activity digital biomarkers, which is a key finding from our work.

We further explored how the ITA model performs in symptomatic versus asymptomatic COVID-19-positive individuals in each cohort. We considered participants to be symptomatic who reported any symptoms in the detection period or on the diagnostic test date. Assuming a diagnostic testing capacity of 30%, ITA indicates testing for 19 of 29 symptomatic and 7 of 21 asymptomatic COVID-19-positive individuals in the cross-validated model using both steps and RHR features, and 5 of 8 symptomatic and 1 of 5 asymptomatic COVID-19-positive individuals in the independent test set of the AF cohort. In other words, 7 of 26 (27%) and 1 of 6 (17%) COVID-19-positive individuals were asymptomatic in the ITA-determined subpopulation for the cross-validated training set and an independent test set of the AF cohort, respectively. Results were similar for the AHF and FHF cohorts (Supplementary Table 5). These findings indicate that the ITA model can not only target diagnostic testing resources toward individuals with symptoms, but also those without any reported symptoms, further increasing the utility of this method.

## Discussion

The COVID-19 pandemic revealed the fragility of our existing healthcare infrastructure to detect the virus and prevent its spread. One key tool for reducing disease spread is bringing diagnostic testing to the right people at the right time and ensuring appropriate interpretation of the diagnostic testing results based on the prevalence of the disease in the population^4^. In light of this need, in April 2020 we developed CovIdentify to integrate commercial wearable device data and electronic symptom surveys to assess the real-time risk of being infected with COVID-19. We envisioned two possible scenarios where CovIdentify would be useful for informing intelligent testing decisions, including (1) ranking individuals in a group by likelihood of current infection with COVID-19 to determine who to test, and (2) tracking a single individual over time for evidence of new infection onset to determine when to test. In our initial development of the ITA model, we focused on the first question, and ultimately improved the positivity rate of COVID-19 diagnostic testing up to 6.5 fold when compared against RTA. These results indicate that if deployed on a large scale, the ITA model could potentially be used to better allocate diagnostic testing resources. To test the real-world efficacy of the ITA model, a simple approach may be to compare the positivity rate of ITA recommended diagnostic testing versus traditional surveillance testing in cohorts of school teachers in the same jurisdiction or school (i.e., similar prevalence rate). This method is likely applicable to other diagnostic areas as well, where digital biomarkers can be used to indicate the likelihood of disease.

In this work, we demonstrated that wearable device data can be used to strategically target the allocation of diagnostic tests to where they are most useful. This approach not only increases testing efficiency and allocation but also reduces the costs and supply chain burden of surveillance testing which is an ongoing challenge. Our results further demonstrate that the ITA method is able to filter a surveillance population to generate a subpopulation with a higher density of true positives, regardless of the prevalence and pretest probability of COVID-19 infection in the population under surveillance for the disease, and, thus, increases testing positivity rates. We note that, although there is a possibility that our COVID-19-negative group may contain other illnesses (e.g., flu) which also reflects a more realistic setting, we were still able to improve resource allocation by over 450% in the independent test set. Another key contribution of our work is the utility of the ITA model using only physiological digital biomarkers (RHR). As steps (and other physical activity) may be reduced due to other reasons than COVID-19 infection, steps may result in nonspecific models, as we have observed from our results on the independent test set. For that reason, an ITA model using more specific digital biomarkers (e.g., RHR) demonstrates the potential of solely relying on physiological data from wearables to develop such an ITA model in a resource-limited setting. We also demonstrate the utility of the ITA to filter individuals for allocating diagnostic tests not only in cases of symptomatic individuals but also for asymptomatic individuals who may not be tested and diagnosed otherwise. While the sensitivity and specificity of diagnostic tests are not affected by ITA, this more efficient testing allocation approach identifies more cases in less time and with fewer resources^41–44^.

The basis of the ITA method is the detection of physiological changes associated with infection onset, which are well established to be detectable by biometric sensors^20–24,26,34–37^. Consistent with prior literature, we demonstrate here that digital biomarkers derived from heart rate and physical activity are indicative of infection onset. A unique contribution of our work is the demonstration of differences in digital biomarker significance with respect to time prior to the diagnostic test date; specifically, we show that differences in RHR features were significant between COVID-19-positive and -negative groups as early as 10 days prior to the diagnostic test date whereas differences in most steps features were not significant until 5 days prior to the diagnostic test date. One steps feature, minimum ΔSteps, was significant up to 9 days prior to the diagnostic test date, potentially demonstrating a link between activity levels (and perhaps noncompliance with lockdown measures) and COVID-19 exposure. Furthermore, RHR begins to deviate from baseline earlier than steps (as early as 13 vs. 10 days prior to the diagnostic test date, respectively), and the peak effect (maximum deviation from baseline) of infection also occurs earlier in RHR than steps (1 day prior vs. 2 days after the diagnostic test date, respectively) for those who were COVID-19 positive. These results indicate that changes in physiology (RHR) occur earlier in the infection period, while symptoms and reduced physical activity (steps) transpire later in the infection period, when people may limit their movement due either to illness or mandatory quarantine. A recent COVID-19 study assessing prolonged physiological and behavioral changes using wearables also observed that COVID-19-positive individuals took more time to return to their RHR baseline values compared to their step and sleep baseline values following the acute COVID-19 infection period^31^; however, this work explored the post-infection period of the data whereas here we explore the pre-infection period as well as the acute infection period using a systematic grid search approach. Another recent study^34^ that developed machine learning models to passively detect COVID-19 using wearable data noted relative changes in feature importance when including data post-diagnosis. However, to our knowledge, we are the first to demonstrate and establish the dynamics of feature importance over time prior to the diagnostic test date, indicating which features should be weighted more heavily in prediction models and when.

Another important contribution of our work is demonstrating the utility of RHR and steps features in the tradeoff between the true-positive rate and false-positive rate (ROC analysis) and the tradeoff between the true-positive rate and the positive predictive value (PR analysis). Specifically, we show that while steps features provide more salient information on the tradeoff between the true-positive rate and false-positive rate, RHR features provide more salient information on the tradeoff between the true-positive rate and the precision (positive predictive value). To our knowledge, this is the first demonstration of this tradeoff in predictive model development for COVID-19 infection detection. The ITA model, in addition to using features of RHR and steps, can likely be further extended and improved with features from other digital biomarkers such as skin temperature, respiratory rate, blood oxygen saturation, and sleep duration^25,26,35,36^. It is anticipated that each of these distinct digital biomarkers would capture a physiological response to infection at different times during the detection period, thus improving the robustness and overall performance of the ITA approach.

One of the important observations from our work was the clear separation of the 95% confidence intervals of the means of digital biomarkers between COVID-19-positive and -negative populations as early as 5 days prior to the test date (Fig. 2a and Supplementary Figs. 6a and 7a), while the variances of the groups have overlapping distributions in the same time window (Supplementary Fig. 8). Notably, a lack of overlap in 95% confidence intervals does not necessarily imply significant differences between the groups^32^ as standard deviation is a valuable descriptive measure of the data that should be considered as well. There are many possible sources of variance in studies involving wearable data, including the inclusion of different device types and technologies, contexts of measurement (e.g., time of day, activity type, etc.), differences in physiological response to infection, etc. We mitigated this issue by segmenting by device type and data resolution, as well as by utilizing measurements during resting periods only for the RHR calculation. In the future, larger datasets can enable segmentation by demographics (e.g., age, sex, weight, etc.) that would likely further reduce the variance. Sharing datasets between studies, as demonstrated here, can also augment the study population and further reduce the variance. An open question is whether the resolution of current photoplethysmography-based wearable heart rate technologies is high enough to adequately detect signals above the population variance.

Here, we did not deploy the ITA method in real-time and, thus, its performance in practice still remains to be tested. Both the CovIdentify and MyPHD studies were primarily bring-your-own-device study designs, in which people who already own smart devices are recruited to participate. The bring-your-own-device design presents two major challenges: (1) participants must own a smart device, which limits eligibility to those who can afford devices, and (2) many different types of devices are used, introducing an additional source of noise in the analysis. We mitigated the first challenge by developing and implementing the Demographic Improvement Guideline, which resulted in a 250% increase in the representation of black and African American participants and a 49% increase in the Latinx and Hispanic population within 4 months of the implementation of the guideline^45^. The second challenge by dividing our overall dataset into cohorts with homogeneous sampling frequencies and/or device types. Although we recognize that certain factors decrease the likelihood of wearable device ownership, such as lower income or living in a rural area^46–48^, the precipitously decreasing cost of wearable technology is rapidly increasing the equitable distribution of these technologies^49^.

Another limitation of the study is the data missingness and its impact on the deviation of the digital biomarkers, as the source of missingness may confound the disease-related physiological variation. For example, we observed that some participants in our study did not wear their devices when they were feeling sick and/or during sleep, as observed in other studies^23^, which resulted in a reduction in data availability as a result of our rigorous data inclusion criteria. For that reason, it can be a challenge to isolate the effects of physiological and behavioral changes on the digital biomarkers. Furthermore, some devices require more frequent charging (e.g., Apple Watch), which results in more missing data that may also impact model performance. We mitigated this challenge by further developing our model on a single device and homogeneous sampling frequency (FHF) cohort.

Another limitation of the study is the self-reported diagnostic testing results from the majority of our study participants. While we acknowledge that self-reported COVID-19 testing results can be less reliable than clinically documented results, similar COVID-19 digital health studies^23,24,27^ utilized self-reported diagnostic testing results for their algorithm development. To instill further confidence in this approach, it is worth noting that if any inaccuracies do exist in the reported testing, which is to be expected in a real-world setting where inaccurate diagnostic testing can occur regularly, our study population was sufficiently large to be powered to handle such noise and variance as demonstrated by the strength of the results.

The recent body of work on COVID-19 detection using smartwatches uses AUC-ROC to evaluate model performance^24,34–37^, which is only an appropriate metric for class-balanced data, and is otherwise misleading^38,39^. In these large-scale studies conducted on a convenience sample of the population for a disease with low prevalence, there exists an inherent challenge of class imbalance because most of the study population does not contract the disease. This was a challenge that we faced in our study, and, further complicating matters, many of the COVID-19-positive participants did not wear their wearable devices at the start of their infection, exacerbating the class imbalance. While less frequently reported than AUC-ROC, the AUC-PR is the correct evaluation metric for evaluating a classifier on imbalanced data^38^, which is what we report here. We show that even with a strong AUC-ROC, the AUC-PR demonstrates the limitations of performance. Methods to resolve class imbalance, especially when working with wearable device data, can be further investigated for future studies. Furthermore, more advanced artificial intelligence methods such as reinforcement learning or graph neural networks may further enhance the performance of the ITA model and is a topic that will be further explored in future studies.

While our study focused on improving testing allocation for COVID-19, the methods developed herein are extensible to other types of infections and could be used to fortify our future pandemic preparedness. Using ITA to improve disease surveillance could be especially important in underserved communities that may benefit from the fact that the ITA method is useful even with only steps digital biomarkers which may be obtained from smartphones which are owned by 85% of the population in the US^50^ and up to 76% globally^51^. By targeting diagnostic testing toward individuals who are more likely to truly be infected with a disease, we can improve the allocation and utility of diagnostic tests, ultimately reducing mortality and increasing our ability to control current and future pandemics.

## Methods

### Participant recruitment and data collection

The CovIdentify study launched on April 2, 2020 (Duke University Institutional Review Board #2020-0412). Eligibility criteria included age over 18 years and internet access. Social networks and social media advertising were used to recruit participants. By May 25, 2021, a total of 7348 participants were recruited and e-consented through the REDCap system^28^. During enrollment, participants were given the option to donate 12 months of retrospective wearable data and 12 months of prospective wearable data. Wearable data was collected via the CovIdentify iOS app for devices connected to the Apple Health kit (e.g., Apple Watch) or via Application Programming Interfaces for other devices (e.g., Garmin and Fitbit devices). The participants were also asked to complete an onboarding (enrollment) survey and daily surveys. The surveys were in English or Spanish and included questions on symptoms, social distancing, diagnostic testing results, and related information (Supplementary Note 1). Surveys were collected using the CovIdentify iOS app, text messaging, and/or emails. All wearable data and survey results were stored in a secured Microsoft Azure data platform and later analyzed in the Microsoft Azure Machine Learning environment. Soon after CovIdentify was launched, exploratory data analysis (EDA) revealed major differences between CovIdentify demographics and the demographics of COVID-19-positive cases and deaths in the U.S., as well as overall U.S. demographics based on the 2020 U.S. Census^52,53^. We sought to mitigate the imbalance throughout the duration of the study by providing wearable devices to underrepresented populations^45^. COVID-19 vaccine reporting was added to the daily surveys in February 2021, where we asked questions regarding the vaccination date, vaccine brand, vaccine-related symptoms, and dose number.

### Wearable data processing and analysis

Participants were asked to fill out an enrollment survey following the informed e-consent. Daily symptom surveys and wearable data from the participants were analyzed both separately and together. For the overall analysis, we only included participants with self-reported diagnostic test results for COVID-19. These participants were further divided into two categories based on the self-reported diagnostic test results: COVID-19 positive and COVID-19 negative.

In addition to the data collected via CovIdentify, we augmented our analysis by including data from the MyPHD study, as reported in the two recent publications by Mishra et al.^23^ and Alavi et al.^27^. The data from Mishra et al. included heart rate, step count, and sleep data for 27 COVID-19-positive cases. It also included metadata of symptom onset and test dates. The data from Alavi et al. included heart rate and step count data for 83 COVID-19-positive cases and 1019 COVID-19-negative cases as well as metadata including symptom onset and test dates.

For wearable data analysis, we only included days of wearable data when both heart rate and step count were available. Out of the 1239 participants (113 from CovIdentify and 1126 from MyPhD study) who had both heart rate and step count data available, we had device-reported daily values of RHR and step count for 67 participants, and high-frequency (second or minute level, depending on device types) wearable data for 1172 participants. For participants with high-frequency heart rate data, we calculated daily RHR from the heart rate data points recorded between midnight and 7 AM, when there were no steps recorded. For those participants with available high-frequency wearable data, we chose a data-driven threshold (i.e., a minimum number of heart rate data points between midnight and 7 AM with zero recorded steps) to include our calculated RHR data from that day in the subsequent analysis. As the sampling rate varies by device type (Fitbit, Garmin, and Apple Watch), we generated separate data distributions of the datasets for these three device types and selected the first quartile of heart rate data points per device as the data-driven threshold, which resulted in a threshold of 2630, 19, and 1389 heart rate data points for Fitbit, Apple Watch, and Garmin devices, respectively. In other words, on a given day, a participant with Fitbit wearable data required at least 2630 heart rate data points between midnight and 7 AM with zero recorded steps to be included in the subsequent analysis. Following this intraday data point threshold, we used an interday data threshold: a minimum number of days with available wearable data to be included in the analysis (50% in the baseline period and 50% between 9 days and 1 day prior to the diagnostic test date in the detection period). We explored different minimum number of days of available wearable data in the baseline and detection periods and selected these two thresholds to maximize the number of participants while keeping the performance of the ITA model on the training dataset consistent, defined as less than 10% variation of the performance metrics (AUC-ROC and AUC-PR)).

### Cohort definition

The wearable data availability thresholds (both intraday and interday) resulted in an AF cohort of 520 participants (83 from CovIdentify and 437 from MyPHD) with sufficient wearable data (63 COVID-19 positive and 457 COVID-19 negative). 24 of the 63 COVID-19 positive cases had clinical documentation for their diagnosis while the others were self reported. We then created two more subsets from this cohort (Supplementary Fig. 3): (1) AHF cohort: participants with high-frequency wearable data (469 participants, 54 COVID-19 positive and 415 COVID-19 negative), and (2) FHF cohort: participants with high-frequency wearable data from a single source (Fitbit) (280 participants, 40 COVID-19 positive and 240 COVID-19 negative) to explore the impact of utilizing wearable data from different sources and resolutions on the ITA model development. We employed these three cohorts separately for the ITA model development and compared the resulting models’ performance in the corresponding training and test datasets of these cohorts. We divided each cohort into an 80% train and 20% test split, with FHF as a subgroup of AHF (which itself is a subset of AF) to ensure that no observations in the training dataset of one cohort existed in the test dataset of another (Supplementary Fig. 3).

### Digital biomarker definition

Given the use of datasets with different device types, a consistent RHR definition was used in order to harmonize the cohorts with high-frequency wearable data. We calculated the daily RHR digital biomarker by aggregating the high-frequency heart rate data points available between midnight and 7 AM, when there were no steps recorded. Step count was calculated by summing all recorded step values during a 24-h period in order to produce a daily step count digital biomarker.

### Feature engineering and extraction

Following the creation of three cohorts (AF, AHF, and FHF) and their corresponding training and test sets, we performed EDA and extracted features from the time-series digital biomarkers (RHR and step count). For the EDA on the time-series digital biomarkers, we explored the difference in trajectories of digital biomarkers between COVID-19-positive and COVID-19-negative participants (Fig. 2a and Supplementary Figs. 6a and 7a). Following the EDA, we extracted the features mentioned in Table 2 from the raw digital biomarkers. We first calculated four deviation metrics, which capture the deviation in digital biomarkers from participants’ baseline during the detection phase. Following the deviation metrics calculation, we calculated summary statistics of these four deviation metrics which we refer as to features for this manuscript. We extracted the same features from the training and test datasets. Following the feature extraction, we performed statistical analysis on the features from the training datasets of the three cohorts to see which features are statistically different between the two groups and how their significance levels vary with different detection period combinations (detection end date and detection window length) using a systematic grid search to optimize detection end date and detection window length (Fig. 2b and Supplementary Figs. 6b and 7b). We utilized multiple hypothesis testing with Benjamini–Hochberg adjusted *p* values for this statistical analysis. Following the statistical analysis and systematic grid search to obtain the optimal detection period to extract the features, we only utilized the intersection of the statistically significant features (*p* value <0.05; unpaired *t*-tests with Benjamini–Hochberg multiple hypothesis correction) extracted from digital biomarkers recorded between 5 days and 1 day and 3 days and 1 day prior to the diagnostic test date for the development of the ITA model.

### ITA model development

Following feature extraction, we developed predictive models to classify COVID-19-positive and -negative participants in the training dataset of each cohort (AF, AHF, and FHF) using nested CV and later validated the models on corresponding independent test datasets. We chose five state-of-the-art machine learning models (logistic regression, K-nearest neighbor, support vector machine, random forest, and extreme gradient boosting^54,55^) for the development of the ITA models to explore how increasing model complexity and adding non-linearity would impact the model performance. We trained these classification models on the training dataset using nested CV with an inner CV loop for hyperparameter tuning and an outer CV loop for model selection. For model training, we selected recall as our preferred scoring metric for model selection to emphasize the relative impact/cost of false negatives compared to false positives, as an individual who is truly positive for COVID-19 and is wrongly classified as negative (or healthy) would further spread disease. For model performance evaluation, we used two well-established reporting metrics, including the most commonly reported metric for studies of this kind (AUC-ROC)^24,33–37^, and the metric that is most appropriate for this classification task (AUC-PR)^38^ (Supplementary Table 3, Figs. 3 and 4, and Supplementary Fig. 10). AUC-PR is more appropriate with class-imbalanced data^38,39^, which is the case here (12–15% COVID-19 positive and 85–88% negative for each of the three cohorts). The results reported for the training dataset (Supplementary Table 3 and Fig. 3a–f) were generated from the validation on the held-out dataset (fold) from each iteration of the outer CV loop which was not used in the model training. Based on the CV results of the five machine learning models on the training dataset, we chose the logistic regression model to further evaluate performance on the independent testing dataset (Supplementary Fig. 10a–f). For validation on the independent test dataset, we trained the logistic regression model on the entire training dataset using a grid search with five stratified folds for hyperparameter tuning and selected the best model (with tuned hyperparameters) to validate on the test dataset.

### Nested cross-validation

For model development with the training dataset, we utilized nested CV over traditional CV, which is a common approach in similar studies^24,34,36,37^, because it uses the same data for hyperparameter tuning and model performance evaluation^56^. In nested CV (also called double CV), the hyperparameter tuning procedure is nested (inner loop) under the model selection procedure (outer loop) and the inner loop is used for optimizing the hyperparameters of the model with inner CV, and the outer loop is used to compute the error of the optimized model with outer CV^57^. For the nested CV, we divided the training set into ten stratified folds (keeping the ratio of COVID-19-positive and -negative participants the same across each fold) for the outer loop. For each iteration of the outer loop, the model was trained on data from nine folds by optimizing the hyperparameters of the model with inner CV, and validating on the left-out fold, a process which was repeated nine more times. In each iteration of the outer loop, the outer training data (from nine folds) were further divided into five stratified folds (inner loop) to tune hyperparameters using a grid search. Out of the five iterations with the grid search in the inner loop, the best model (including hyperparameters) was selected, and this model was used in the model performance evaluation in the outer loop. This way of model development using two CV steps separates hyperparameter tuning and model selection in order to reduce bias in model performance.

### Feature importance ranking

To calculate the feature importance ranking, we trained the logistic regression model using a grid search with five stratified folds for hyperparameter tuning and selected the best model (with optimized hyperparameters) to train on the entire training set of each cohort, and extracted the coefficients for each feature used in the optimized model. We reported the absolute value of each coefficient as the relative importance of the features (Supplementary Fig. 12).

### Reporting summary

Further information on research design is available in the Nature Research Reporting Summary linked to this article.

## Supplementary information

Supplementary Figures and Tables

Supplementary Note 1

Reporting Summary

## Data Availability

The de-identified CovIdentify dataset generated and/or analyzed during the current study will be submitted 1 year from the publication date to the Digital Health Data Repository (DHDR) repository (https://github.com/DigitalBiomarkerDiscoveryPipeline/Digital_Health_Data_Repository) under the title BigIdeasLab_CovIdentify. The de-identified MyPHD dataset used in Alavi et al. (*Nature Medicine* 2021) study can be downloaded at the following publicly available link: https://storage.googleapis.com/gbsc-gcp-project-ipop_public/COVID-19/COVID-19-Wearables.zip and the dataset used in Mishra et al. (*Nature Biomedical Engineering* 2020) study can be downloaded at the following publicly available link: https://storage.googleapis.com/gbsc-gcp-project-ipop_public/COVID-19-Phase2/COVID-19-Phase2-Wearables.zip.

## Code availability

ITA model development code used for this manuscript is available on the digital biomarker discovery pipeline (DBDP) GitHub repository (https://github.com/DigitalBiomarkerDiscoveryPipeline/CovIdentify).
