# Supplementary material for: A method for intelligent allocation of diagnostic testing by leveraging data from commercial wearable devices: a case study on COVID-19
Source: NPJ Digit Med. 2022 Sep 1;5:130. doi: 10.1038/s41746-022-00672-z (PMC9434073; doi:10.1038/s41746-022-00672-z)
Supplement: Supplementary file 1 — Supplementary Figures and Tables [file 41746_2022_672_MOESM1_ESM.pdf]

Supplementary Figure 1

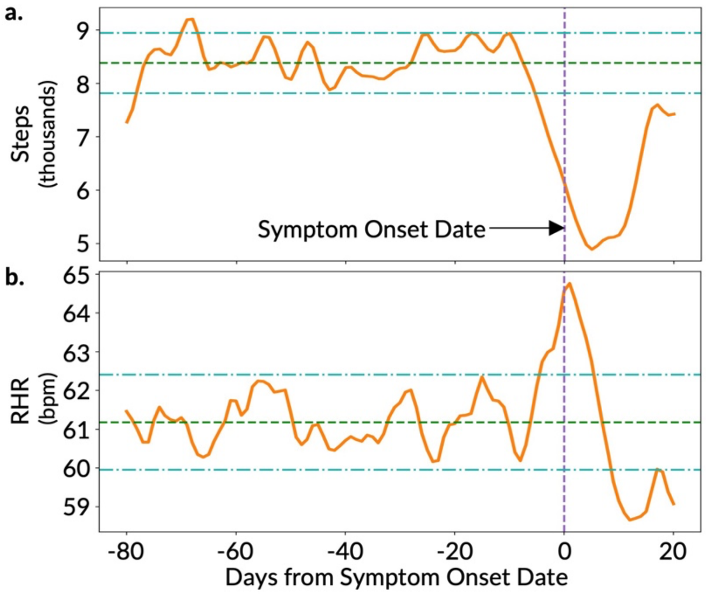

Supplementary Figure 1. Time-series plot of digital biomarkers with respect to symptom onset date. (a) Time-averaged step count and **(b)** time-averaged resting heart rate (RHR) of the subset of participants (N=33) in the training set with available symptom onset date who tested positive for COVID-19. The horizontal green dashed lines and the horizontal light green dash-dotted lines show the baseline period mean and ± 2 standard deviations from the baseline mean respectively.

Supplementary Figure 2

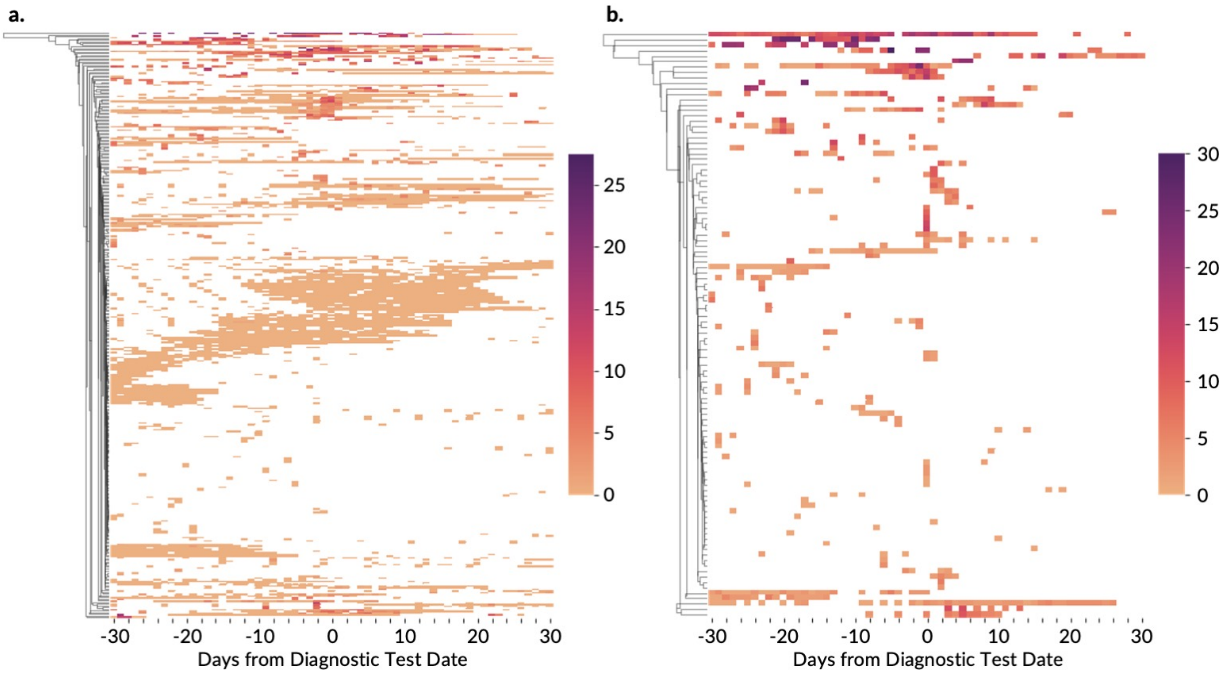

Supplementary Figure 2. Hierarchical clustering of COVID-19 negative participants.
a. CovIdentify participants (n = 348) **b.** MyPHD participants (n =108). Each participant is represented by a row on the heatmap to the right of the dendrogram. The color scale demonstrates the daily sum of the symptom ratings, where each symptom ranged on a scale from 1 to 5 (purple = most symptomatic and peach = least symptomatic). Areas in white show where no symptoms were reported.

Supplementary Figure 3

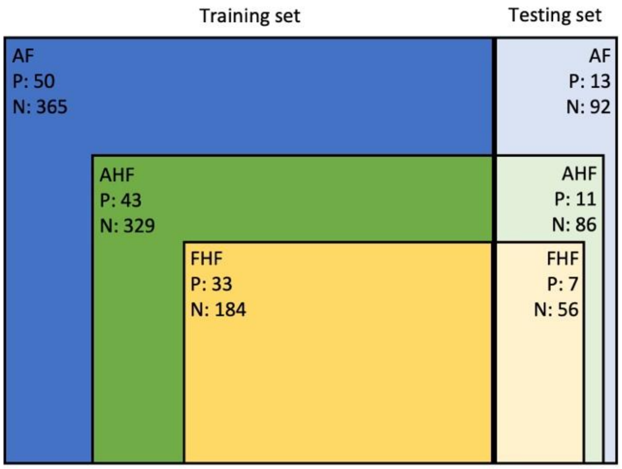

Supplementary Figure 3. Cohort definitions. All three cohorts (All Frequency, AF (blue), All-High-Frequency, AHF (green), and Fitbit-High-Frequency, FHF (yellow)) were divided into an 80% train and 20% test split, with FHF as a subgroup of AHF, which itself is a subset of AF to ensure that no observations in the training set of one cohort existed in the test set of another.

Supplementary Figure 4

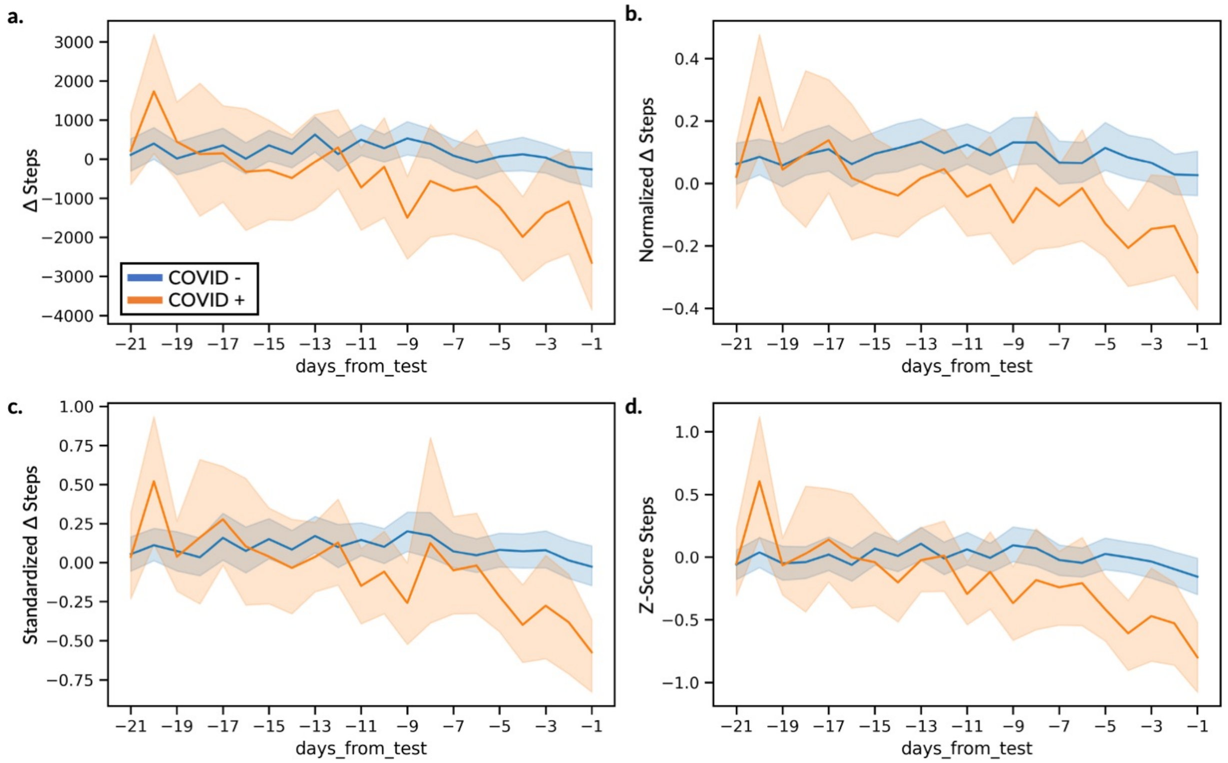

Supplementary Figure 4. Time-series plot of four deviation metrics from the steps digital biomarker for the AF cohort. (a) deviation in step count from baseline median (detection – baseline median), ΔSteps, **(b)** normalized ΔSteps, **(c)** standardized ΔSteps, and **(d)** z-score ((detection – baseline mean) / baseline standard deviation) of Steps between the participants diagnosed as COVID-19 positive and negative. Confidence bands show the 95% confidence intervals.

Supplementary Figure 5

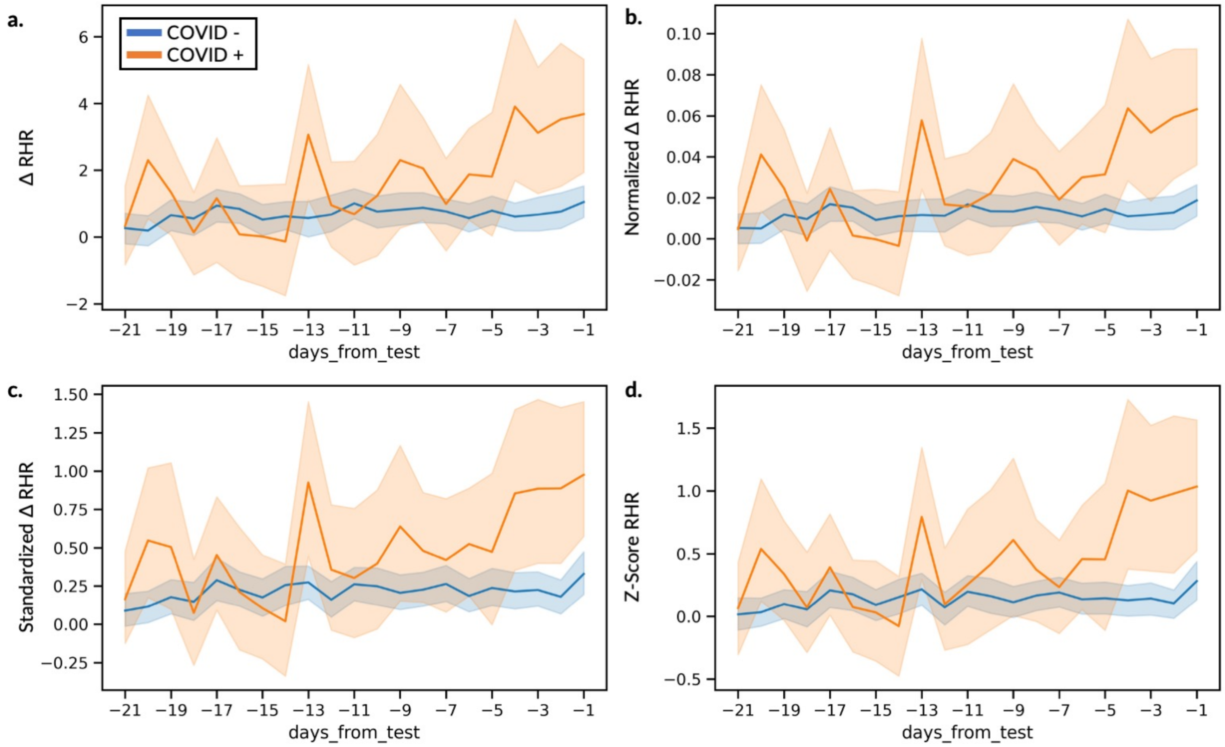

Supplementary Figure 5. Time-series plot of four deviation metrics from the RHR digital biomarker for the AF cohort. (a) deviation in RHR from baseline median (detection – baseline median), ΔRHR, **(b)** normalized ΔRHR, **(c)** standardized ΔRHR, and **(d)** z-score ((detection – baseline mean) / baseline standard deviation) of RHR between the participants diagnosed as COVID-19 positive and negative. Confidence bands are showing the 95% confidence intervals.

Supplementary Figure 6

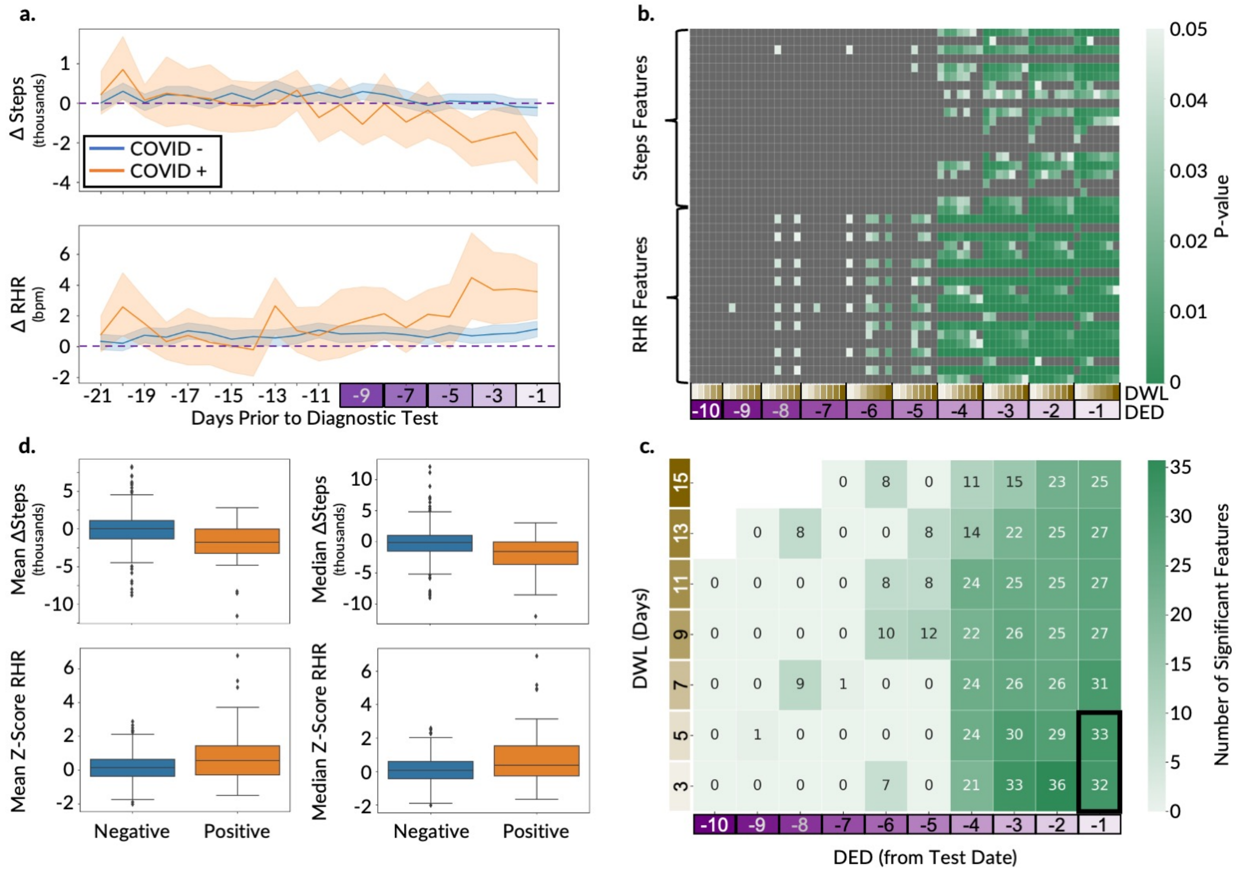

Supplementary Figure 6. Overview of the feature exploration and extraction for the ITA model development on the AHF cohort. a. Time-series plot of the deviation in digital biomarkers (ΔSteps and ΔRHR) in the detection window compared to baseline periods, between the participants diagnosed as COVID-19 positive and negative. The horizontal dashed line displays the baseline median and the confidence bounds show the 95% confidence intervals. **b.** Heatmaps of steps and RHR features that are statistically significantly different (p-value < 0.05, unpaired t-tests) in a grid search with different DED and DWL combinations, with green boxes showing p-values < 0.05 and gray boxes showing p-values ≥ 0.05. The p-values are adjusted with the Benjamini-Hochberg method for multiple hypothesis correction. **c.** Summary of the significant features (p-value < 0.05, unpaired t-tests) from B, with each box showing the number of statistically significant features for the different combinations of DED and DWL. The intersection of the significant features across DWL of 3 and 5 days with a common DED of 1 day prior to the test date (as shown using the black rectangle) were used for the ITA model development. **d.** Box plots comparing the distribution of the two most significant steps and RHR features between the participants diagnosed as COVID-19 positive and negative. The centerlines denote feature medians, bounds of boxes represent 25th and 75th percentiles, whiskers denote nonoutlier data range and the diamonds denote outlier values.

Supplementary Figure 7

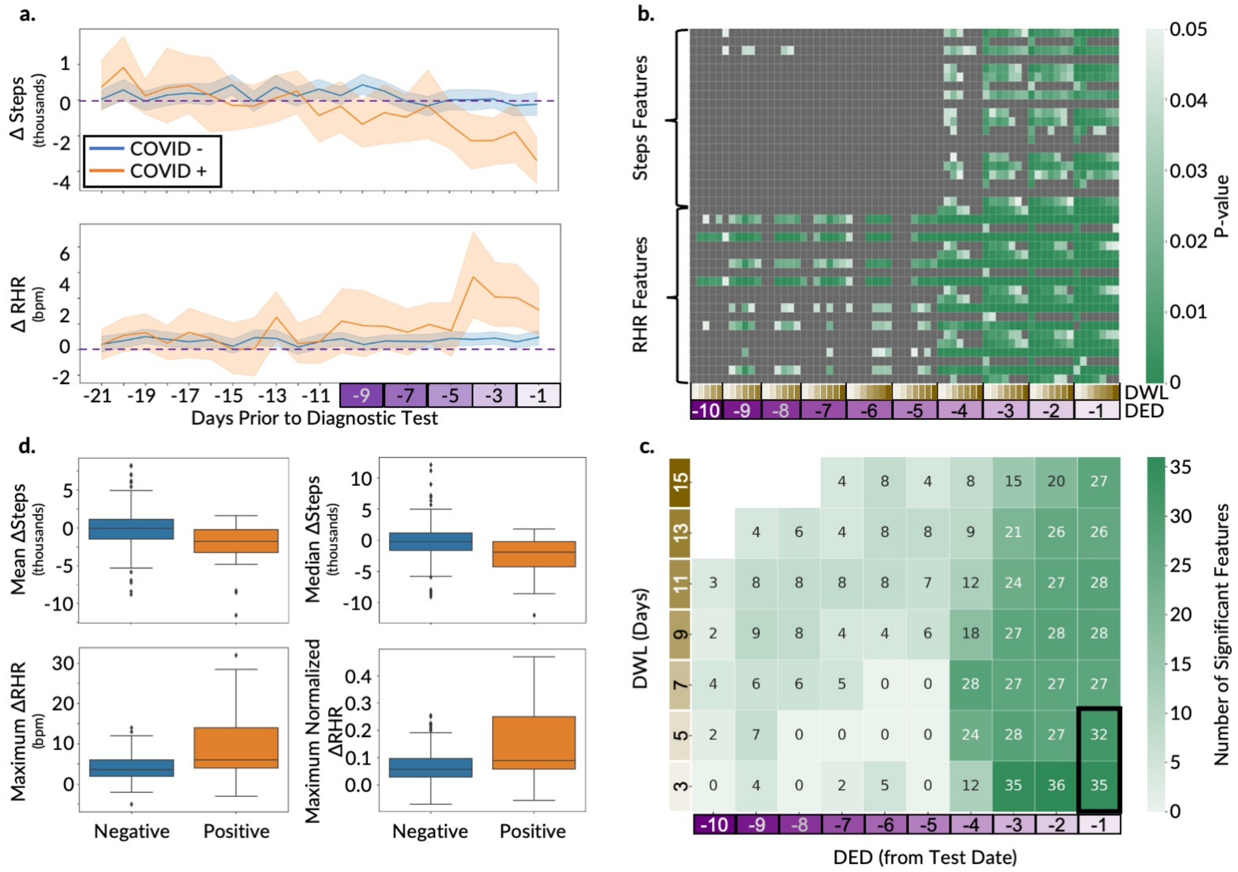

Supplementary Figure 7. Overview of the feature exploration and extraction for the ITA model development on the FHF cohort. a. Time-series plot of the deviation in digital biomarkers (ΔSteps and ΔRHR) in the detection window compared to baseline periods, between the participants diagnosed as COVID-19 positive and negative. The horizontal dashed line displays the baseline median and the confidence bounds show the 95% confidence intervals. **b.** Heatmaps of steps and RHR features that are statistically significantly different (p-value < 0.05, unpaired t-tests) in a grid search with different DED and DWL combinations, with green boxes showing p-values < 0.05 and gray boxes showing p-values ≥ 0.05. The p-values are adjusted with the Benjamini-Hochberg method for multiple hypothesis correction. **c.** Summary of the significant features (p-value < 0.05, unpaired t-tests) from B, with each box showing the number of statistically significant features for the different combinations of DED and DWL. The intersection of the significant features across DWL of 3 and 5 days with a common DED of 1 day prior to the test date (as shown using the black rectangle) were used for the ITA model development. **d.** Box plots comparing the distribution of the two most significant steps and RHR features between the participants diagnosed as COVID-19 positive and negative. The centerlines denote feature medians, bounds of boxes represent 25th and 75th percentiles, whiskers denote nonoutlier data range and the diamonds denote outlier values.

Supplementary Figure 8

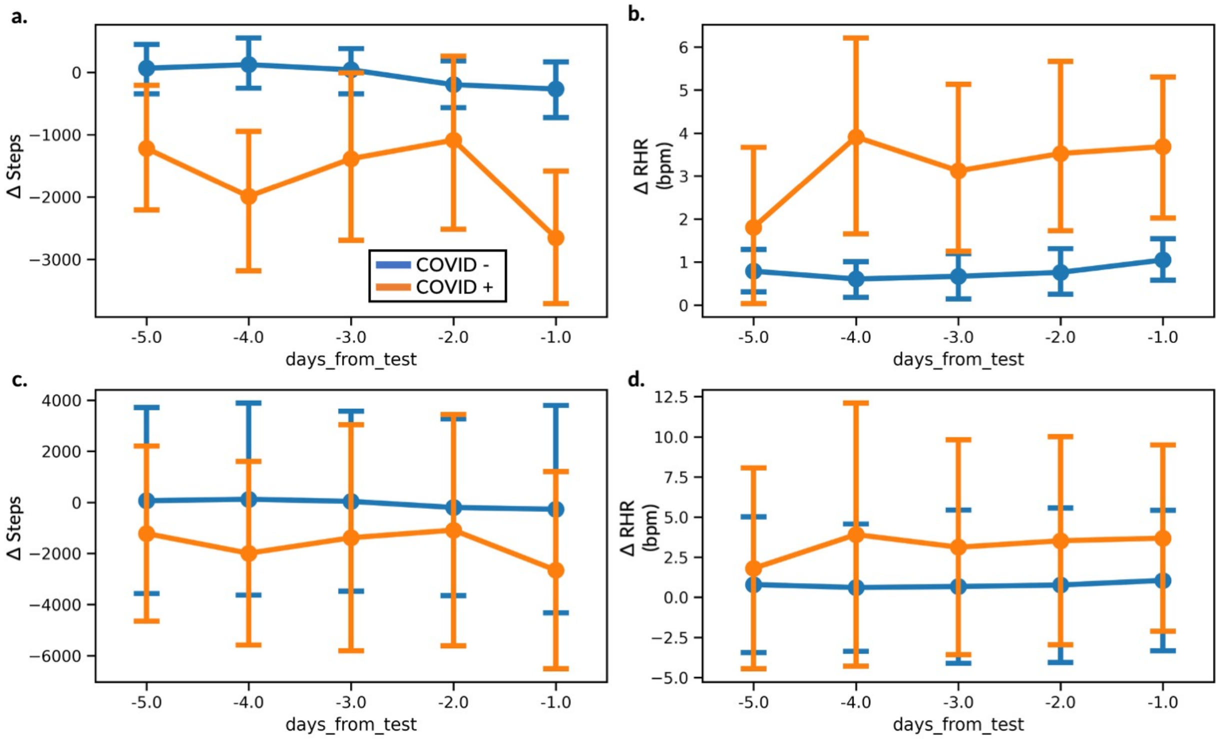

Supplementary Figure 8. Overview of the digital biomarkers in the detection period showing different representations of error bars. Time-series plot of Δ Steps **(a, c)**, and Δ RHR **(b, d)** between the participants diagnosed as COVID-19 positive (orange) and negative (blue). Error bars in A and B show the 95% confidence intervals, whereas error bars in C and D show the one standard deviation.

Supplementary Figure 9

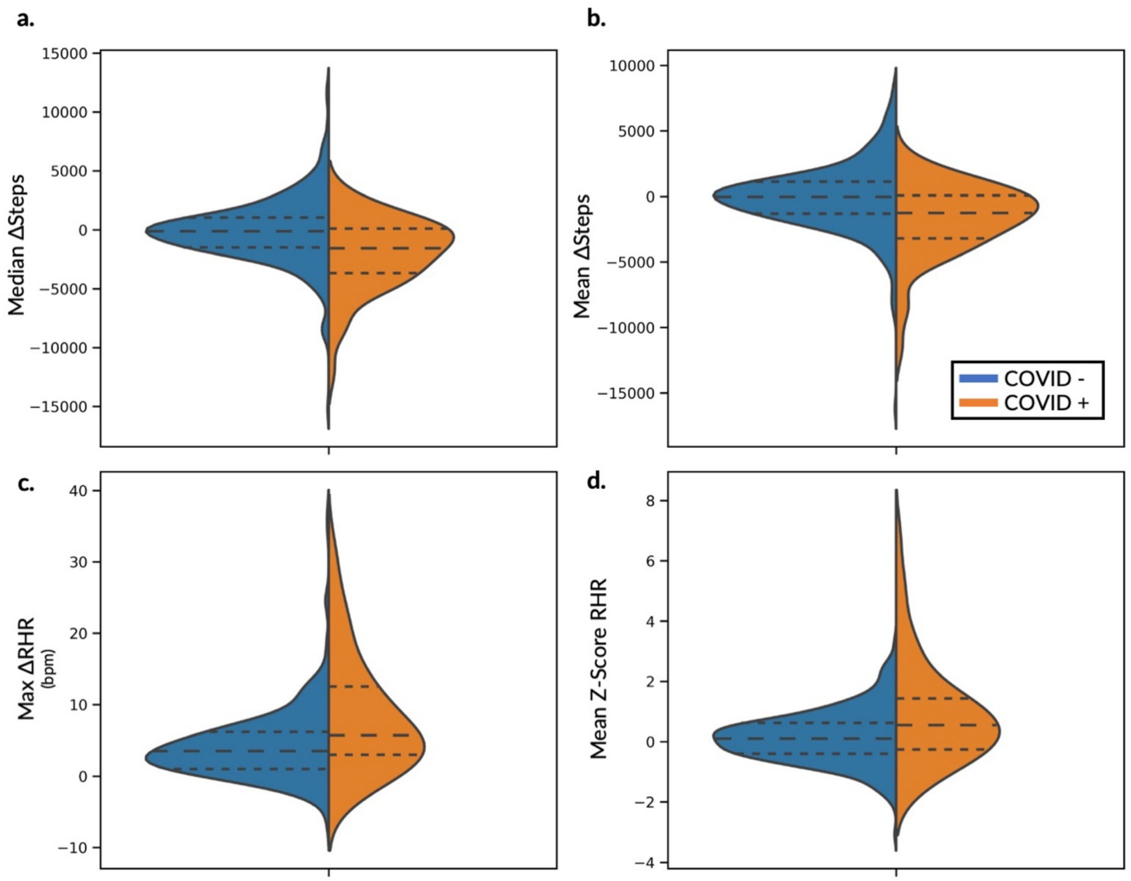

Supplementary Figure 9. Distribution of the top two steps and RHR features across COVID-19 positive and negative groups in the AF cohort. Violin plots of two steps **(a, b)** and RHR **(c, d)** features with the lowest p-values, showing the distribution of the features between the participants diagnosed as COVID-19 positive (orange) and negative (blue). The outer dashed lines represent the first and third quartiles and the inner dashed line represents the median.

Supplementary Figure 10

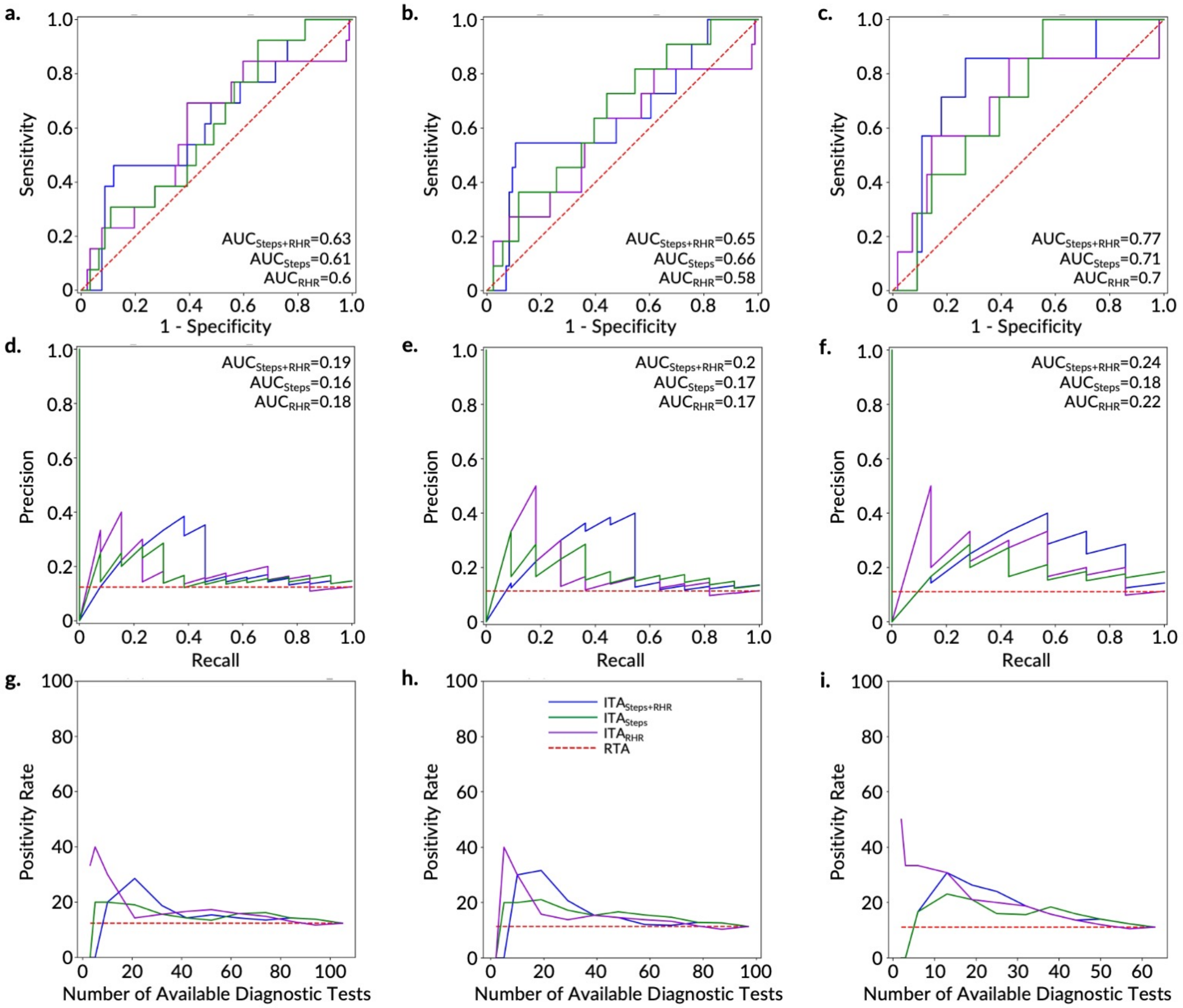

Supplementary Figure 10. Prediction and ranking results of the ITA models on the test sets for the AF (a, d, and g), AHF (b, e, and h), and FHF (c, f, and i) cohorts using features from combination of Steps and RHR (blue), Steps (green), and RHR (violet) digital biomarkers. a-c. ROCs and **d-f.** PRCs for the discrimination between COVID-19 positive participants and negative participants in the test set. The red dashed line shows the results based on an RTA model (the null model). **g-i.** The positivity rate of the diagnostic testing subpopulation as determined by ITA given a specific number of available diagnostic tests. The red dashed line displays the positivity rate/pre-test probability of an RTA (null) model.

Supplementary Figure 11

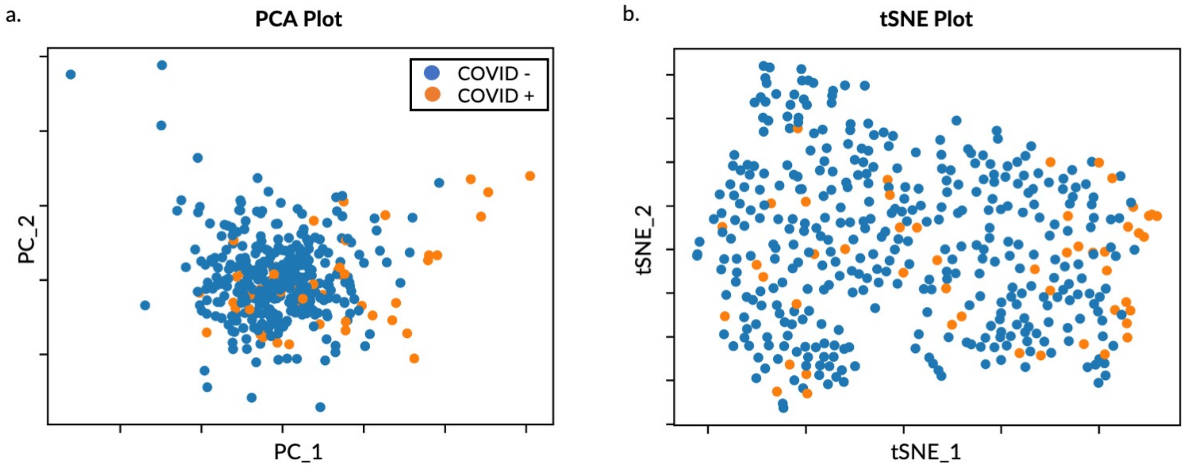

Supplementary Figure 11. Low dimensional representation of input variables for the ITA model using the AF cohort. (a) Principal component analysis (PCA) and **(b)** t-stochastic neighborhood embedding (tSNE) plots for the input variables (significant steps and RHR features).

Supplementary Figure 12

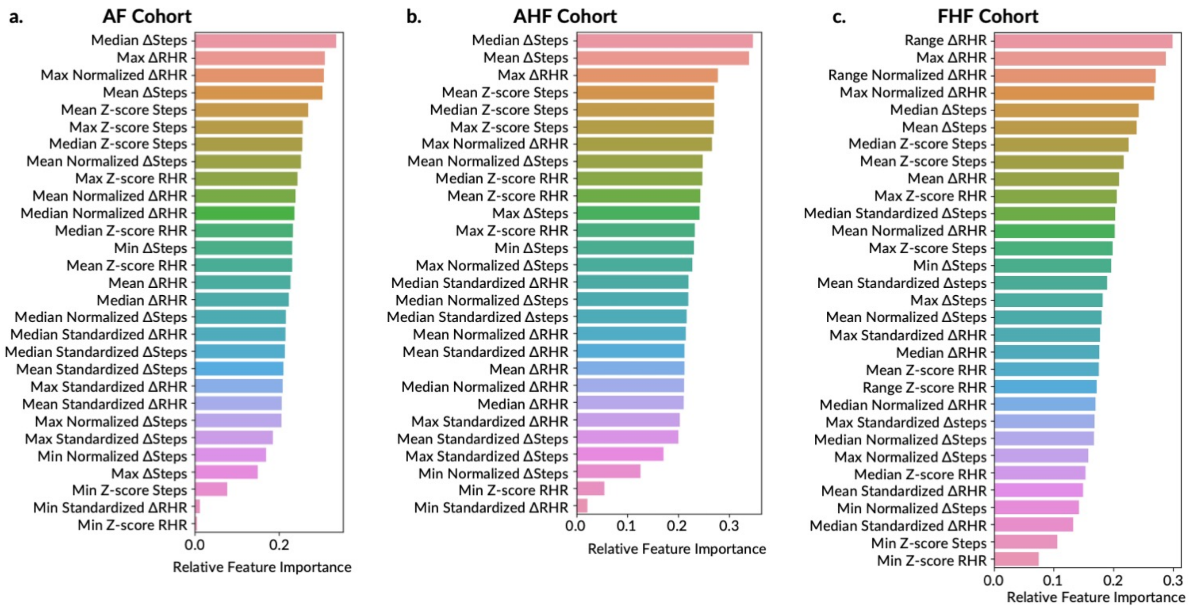

Supplementary Figure 12. Feature importance ranking for the logistic regression models. Feature importance ranking based on the absolute value of coefficients of each feature used in the logistic regression model using training data from the **(a)** AF **(b)** AHF, and **(c)** FHF cohorts. Max, maximum; Min, minimum.

Supplementary Table 1: Demographic information for participants in the CovIdentify study

Total	Completed Enrollment
Demographic data	
Number of participants	6,765
Female	3,841
Race	
White	4,660
Black	251
Asian	215
Alaska Native	26
Pacific Islander	6
Ethnicity	
Not Hispanic	5,591
Hispanic	305
Sensor Ownership	
Fitbit	1,091
Apple Watch	107
Garmin	1,689
Age	
18-29	603
30-39	1,131
40-49	1,459
50-59	1,639
60-69	1,311
>69	597

Supplementary Table 2: Demographic information for participants in the three cohorts

	AF	AHF	FHF
Age			
Age min	19	19	19
Age max	84	81	81
Sex			
Number of females	281	265	184
Ethnicity			
European, Caucasian, White	371	359	231
Black or African-American	13	12	6
Native American or Alaska Native	1	1	1
Asian	15	15	8
Native Hawaiian or Pacific Islander	0	0	0
Hispanic	13	13	6
Other or Combination	24	24	15
I'd prefer not to say	2	2	2
BMI			
BMI less than 25	161	161	103
BMI 25 to 35	197	197	125
BMI greater than 35	35	35	25

Supplementary Table 3: Significant features across three cohorts ordered in an ascending order based on significance (p-value, unpaired t-tests with Benjamini-Hochberg multiple hypothesis correction).

**AF Cohort**	**AHF Cohort**	**FHF Cohort**
Mean Z-score RHR	Median Z-score RHR	Max ΔRHR
Max ΔRHR	Mean Z-score RHR	Max Normalized ΔRHR
Max Normalized ΔRHR	Max Z-score RHR	Range ΔRHR
Median Z-score RHR	Median Standardized ΔRHR	Range Normalized ΔRHR
Max Z-score RHR	Mean Standardized ΔRHR	Max Z-score RHR
Mean Normalized ΔRHR	Max Normalized ΔRHR	Mean Normalized ΔRHR
Mean Standardized ΔRHR	Max ΔRHR	Mean ΔRHR
Median Normalized ΔRHR	Mean ΔSteps	Max Standardized ΔRHR
Mean Standardized ΔRHR	Median ΔSteps	Mean Z-score RHR
Median ΔSteps	Mean Normalized ΔRHR	Mean ΔSteps
Mean ΔRHR	Median Normalized ΔRHR	Median ΔSteps
Max Standardized ΔRHR	Mean ΔRHR	Median Normalized ΔRHR
Median ΔRHR	Max Standardized ΔRHR	Median ΔRHR
Mean ΔSteps	Median ΔRHR	Median Z-score Steps
Mean Z-score Steps	Mean Z-score Steps	Mean Z-score Steps
Median Z-score Steps	Median Z-score Steps	Median Z-score RHR
Mean Normalized ΔSteps	Max Z-score Steps	Mean Standardized ΔRHR
Mean Standardized ΔSteps	Mean Normalized ΔSteps	Range Z-score RHR
Median Standardized ΔSteps	Median Standardized ΔSteps	Median Standardized ΔSteps

Max Z-score Steps	Mean Standardized ΔSteps	Mean Standardized ΔSteps
Median Normalized ΔSteps	Max ΔSteps	Median Standardized ΔRHR
Min ΔSteps	Min ΔSteps	Min ΔSteps
Max Standardized ΔSteps	Median Normalized ΔSteps	Max Z-score Steps
Max Normalized ΔSteps	Max Normalized ΔSteps	Mean Normalized ΔSteps
Min Normalized ΔSteps	Max Standardized ΔSteps	Max ΔSteps
Max ΔSteps	Min Z-score RHR	Max Standardized ΔSteps
Min Z-score RHR	Min Standardized ΔRHR	Median Normalized ΔSteps
Min Standardized ΔRHR	Min Normalized ΔSteps	Min Normalized ΔSteps
Min Z-score Steps		Max Normalized ΔSteps
		Min Z-score Steps
		Min Z-score RHR

Max, Maximum; Min, Minimum.

Supplementary Table 4: Performance of the five machine learning algorithms tested for the ITA model.

Cohort	AF		AHF		FHF	
Model	AUC-ROC	AUC-PR	AUC-ROC	AUC-PR	AUC-ROC	AUC-PR
Logistic Regression	0.69±0.10	0.42±0.18	0.70±0.14	0.42±0.22	0.73±0.12	0.55±0.21
K-Nearest Neighbor	0.61±0.11	0.32±0.17	0.61±0.11	0.32±0.18	0.67±0.12	0.48±0.21
Support Vector Machine	0.63±0.12	0.27±0.17	0.64±0.13	0.32±0.20	0.64±0.16	0.45±0.16
Random Forest	0.67±0.13	0.38±0.17	0.68±0.15	0.41±0.20	0.71±0.14	0.5±0.16
Extreme Gradient Boosting	0.67±0.14	0.35±0.19	0.71±0.12	0.4±0.20	0.71±0.13	0.45±0.20

Results reported in this table are generated from the model developed on the training set of the three cohorts using 10-fold nested cross-validation. The cross-validation results are calculated based on the validation on the left-out-fold in each iteration of the outer loop of the nested cross-validation and are reported in the mean (± SD) format.

Supplementary Table 5: ITA model performance for symptomatic and asymptomatic individuals.

Cohorts	Total Dataset				ITA Determined Subpopulation (30% Testing Capacity)			
	Total N	Total COVID+	Total COVID+ Symptomatic	Total COVID+ Asymptomatic	Total N	Total COVID+	Total COVID+ Symptomatic	Total COVID+ Asymptomatic
**Training Set**								
**AF**	415	50	29	21	124	26	19	7
**AHF**	372	43	29	14	112	25	20	5
**FHF**	217	33	21	12	65	20	16	4
**Test Set**								
**AF**	105	13	8	5	32	6	5	1
**AHF**	97	11	8	3	29	6	5	1
**FHF**	63	7	6	1	19	5	4	1
