# Supplementary material for: A method for intelligent allocation of diagnostic testing by leveraging data from commercial wearable devices: a case study on COVID-19
Source: NPJ Digit Med. 2022 Sep 1;5:130. doi: 10.1038/s41746-022-00672-z (PMC9434073; doi:10.1038/s41746-022-00672-z)
Supplement: Supplementary file 3 — Reporting Summary [file 41746_2022_672_MOESM3_ESM.pdf]

nature research

Corresponding author(s): Jessilyn Dunn

Last updated by author(s): Jul 29, 2022

Reporting Summary

Nature Research wishes to improve the reproducibility of the work that we publish. This form provides structure for consistency and transparency in reporting. For further information on Nature Research policies, see our Editorial Policies and the Editorial Policy Checklist.

Statistics

For all statistical analyses, confirm that the following items are present in the figure legend, table legend, main text, or Methods section.

n/a	Confirmed	
☐	☒	The exact sample size (*n*) for each experimental group/condition, given as a discrete number and unit of measurement
☐	☒	A statement on whether measurements were taken from distinct samples or whether the same sample was measured repeatedly
☐	☒	The statistical test(s) used AND whether they are one- or two-sided<br>*Only common tests should be described solely by name; describe more complex techniques in the Methods section.*
☐	☒	A description of all covariates tested
☐	☒	A description of any assumptions or corrections, such as tests of normality and adjustment for multiple comparisons
☐	☒	A full description of the statistical parameters including central tendency (e.g. means) or other basic estimates (e.g. regression coefficient) AND variation (e.g. standard deviation) or associated estimates of uncertainty (e.g. confidence intervals)
☐	☒	For null hypothesis testing, the test statistic (e.g. *F*, *t*, *r*) with confidence intervals, effect sizes, degrees of freedom and *P* value noted<br>*Give P values as exact values whenever suitable.*
☒	☐	For Bayesian analysis, information on the choice of priors and Markov chain Monte Carlo settings
☒	☐	For hierarchical and complex designs, identification of the appropriate level for tests and full reporting of outcomes
☒	☐	Estimates of effect sizes (e.g. Cohen's *d*, Pearson's *r*), indicating how they were calculated

Our web collection on statistics for biologists contains articles on many of the points above.

Software and code

Policy information about availability of computer code

Data collection	The CovIdentify study launched on April 2, 2020 (Duke University Institutional Review Board #2020-0412). Eligibility criteria included age over 18 years and internet access. Social networks and social media advertising were used to recruit participants. By May 25, 2021, a total of 7,348 participants were recruited and e-consented through the Research Electronic Data Capture (REDCap) system. During enrollment, participants were given the option to donate 12 months of retrospective wearable data and 12 months of prospective wearable data. Wearable data was collected via the CovIdentify iOS app for devices connected to the Apple Health kit (e.g., Apple Watch) or via Application Programming Interfaces (APIs) for other devices (e.g., Garmin and Fitbit devices). The participants were also asked to complete an onboarding (enrollment) survey and daily surveys. The surveys were in English or Spanish and included questions on symptoms, social distancing, diagnostic testing results, and related information (Supplementary Document 1). Surveys were collected using the CovIdentify iOS app, text messaging, and/or emails. All wearable data and survey results were stored in a secured Microsoft Azure data platform and later analyzed in the Microsoft Azure Machine Learning environment.
Data analysis	Analyses were performed using python 3.8.1. The Python packages pandas version 1.3.5 and numpy version 1.19.1 have been used for data processing. Model development and performance evaluation were performed using the Python package scikit-learn version 1.0.1. Statistical tests and p-values have been evaluated using the Python package scipy version 1.5.2. Results were visualized using the Python matplotlib package version 3.2.1 and seaborn package version 0.11.1.<br><br>ITA model development code used for this manuscript is available on the digital biomarker discovery pipeline (DBDP)'s GitHub repository (https://github.com/DigitalBiomarkerDiscoveryPipeline/CovIdentify).

For manuscripts utilizing custom algorithms or software that are central to the research but not yet described in published literature, software must be made available to editors and reviewers. We strongly encourage code deposition in a community repository (e.g. GitHub). See the Nature Research guidelines for submitting code & software for further information.

Data

Policy information about availability of data

All manuscripts must include a data availability statement. This statement should provide the following information, where applicable:

- Accession codes, unique identifiers, or web links for publicly available datasets
- A list of figures that have associated raw data
- A description of any restrictions on data availability

The de-identified CovIdentify data set generated and/or analyzed during the current study will be submitted one year from the publication date to the Digital Health Data Repository (DHDR) repository (https://github.com/DigitalBiomarkerDiscoveryPipeline/Digital_Health_Data_Repository) under the title BigIdeasLab_CovIdentify.

The de-identified MyPHD dataset used in Alavi et al. (Nature Medicine 2021) study can be downloaded at the following publicly available link: https://storage.googleapis.com/gbsc-gcp-project-ipop_public/COVID-19/COVID-19-Wearables.zip and the dataset used in Mishra et al. (Nature Biomedical Engineering 2020) study can be downloaded at the following publicly available link: https://storage.googleapis.com/gbsc-gcp-project-ipop_public/COVID-19-Phase2/COVID-19-Phase2-Wearables.zip.

Field-specific reporting

Please select the one below that is the best fit for your research. If you are not sure, read the appropriate sections before making your selection.

☒ Life sciences ☐ Behavioural & social sciences ☐ Ecological, evolutionary & environmental sciences

For a reference copy of the document with all sections, see nature.com/documents/nr-reporting-summary-flat.pdf

Life sciences study design

All studies must disclose on these points even when the disclosure is negative.

Sample size

A total of 7,348 participants e-consented to the CovIdentify study between April 2, 2020 and May 25, 2021 through the secure research electronic data capture (REDCap) system. Of those consented, 6,765 participants enrolled in the study (Supplementary Table 1) by completing an enrollment survey consisting of 37-61 questions that followed branching logic (Supplementary Document 1). Of those enrolled, 2,887 participants connected their smartwatches to the CovIdentify platform, including 1,689 Garmin, 1,091 Fitbit, and 107 Apple smartwatches. Throughout the course of the study, 362,108 daily surveys were completed by 5,859 unique participants, with a mean of 62 and a median of 37 daily surveys completed per individual. Of all CovIdentify participants, 1,289 participants reported at least one diagnostic test result for COVID-19 (132 positive and 1,157 negative) (Fig 1B). All survey and device data collected through CovIdentify was transferred securely to a protected cloud environment for further analysis. Out of the 1,289 participants with self-reported diagnostic test results, 136 participants (16 positive and 120 negative) had smartwatch data available during the time periods needed for analysis. Due to the observational nature of the study, we did not perform any statistical analysis to predetermine the sample size, which was enforced by the number of active participants enrolled.

To increase the study population size, we augmented our dataset with data from the MyPHD study (Mishra et al. 2020 Nature BME and Alavi et al. 2021 Nature Med). From the MyPHD study, smartwatch, symptom, and diagnostic testing data from an additional 1,129 participants (110 positive and 1,019 negative) were included in this analysis.

Data exclusions

For the overall analysis, we only included participants with self-reported diagnostic test results for COVID-19. For wearable data analysis, we only included days of wearable data when both heart rate and step count were available. Out of the 1,239 participants (113 from CovIdentify and 1,126 from MyPhD study) who had both heart rate and step count data available, we had device-reported daily values of RHR and step count for 67 participants, and high frequency (second- or minute- level, depending on device types) wearable data for 1,172 participants. For participants with high frequency heart rate data, we calculated daily RHR from the heart rate data points recorded between midnight and 7 AM, when there were no steps recorded. For those participants with available high frequency wearable data, we chose a data-driven threshold (i.e., a minimum number of heart rate data points between midnight and 7 AM with zero recorded steps) to include our calculated RHR data from that day in the subsequent analysis. As the sampling rate varies by device types (Fitbit, Garmin, and Apple Watch), we generated separate data distributions of the datasets for these three device types and selected the first quartile of heart rate data points per device as the data-driven threshold, which resulted in a threshold of 2,630, 19, and 1,389 heart rate data points for Fitbit, Apple Watch, and Garmin devices, respectively. In other words, on a given day, a participant with Fitbit wearable data needed to have at least 2,630 heart rate data points between midnight and 7 AM with zero recorded steps for us to include our calculated RHR value in the subsequent analysis. Following this intraday data point threshold, we used an interday data threshold: a minimum number of days with available wearable data to be included in the analysis (50% in the baseline period and 50% between nine days and one day prior to the diagnostic test date in the detection period). We explored different minimum number of days of available wearable data in the baseline and detection periods and selected these two thresholds to maximize the number of participants while keeping the performance of the ITA model on the training dataset consistent, defined as less than 10% variation of the performance metrics (AUC-ROC and AUC-PR)). No data samples that were eligible for the analysis has been excluded.

Replication

This was an observational study in which we did not perform any replication.

Randomization

The wearable data availability thresholds (both intraday and interday) resulted in a cohort (All Frequency (AF)) of 520 participants (83 from CovIdentify and 437 from MyPHD) with sufficient wearable data. We then created two more subsets from this cohort (Extended Data Fig 2): (1) AHF cohort: participants with high frequency wearable data (469 participants, 54 COVID-19 positive and 415 COVID-19 negative), and (2)

	FHF cohort: participants with high frequency wearable data from a single source (Fitbit) (280 participants, 40 COVID-19 positive and 240 COVID-19 negative) to explore the impact of utilizing wearable data from different sources and resolutions on the ITA model development. We employed these three cohorts separately for the ITA model development and compared the resulting models' performance in the corresponding training and test datasets of these cohorts. We divided each cohort into an 80% train and 20% test split randomly, with FHF as a subgroup of AHF (which itself is a subset of AF) to ensure that no observations in the training dataset of one cohort existed in the test dataset of another (Extended Data Fig 2).
Blinding	The cohort and group allocation were solely related to the data shared by the participants. Due to the observational nature of the study, the investigators were not involved in any arbitrary group allocation.

Reporting for specific materials, systems and methods

We require information from authors about some types of materials, experimental systems and methods used in many studies. Here, indicate whether each material, system or method listed is relevant to your study. If you are not sure if a list item applies to your research, read the appropriate section before selecting a response.

Materials & experimental systems

n/a	Involved in the study	
☒	☐	Antibodies
☒	☐	Eukaryotic cell lines
☒	☐	Palaeontology and archaeology
☒	☐	Animals and other organisms
☐	☒	Human research participants
☒	☐	Clinical data
☒	☐	Dual use research of concern

Methods

n/a	Involved in the study	
☒	☐	ChIP-seq
☒	☐	Flow cytometry
☒	☐	MRI-based neuroimaging

Human research participants

Policy information about studies involving human research participants

Population characteristics	For 6,765 participants enrolled (i.e., completed enrollment survey) in the CovIdentify study, the average age was 50.86 years (range, 18-94). Women represented 55.4% of the population. The race distribution of the enrolled cohort was 86% White, 7.1% Black, 4.4% Asian, and 2.5% Mixed/Other/Undeclared. The most common wearable devices were Garmin, Fitbit, and Apple Watches.<br><br>For the MyPHD study dataset from Mishra et al. (Nature BME 2020), the mean age of the 5,262 participants at time of enrollment was 44 (range, 18–88); 55.3% were women. However, out of the 32 individuals analyzed, 25 (78.1%) were women. The self-reported ethnic distribution of the full cohort was 74.9%<br>European, 3.9% East Asian, 2.9% African American, 19.2% Mixed/Other/Undeclared.<br><br>For the MyPHD study dataset from Alavi et al. (Nature Medicine 2021), in total, 3,318 adult individuals (55.8% female, 79% White, 4.6% Asian, 3.6% Hispanic, 3% Black, and 10.6% Mixed/Other/Undeclared) 18–80 years of age were recruited.
Recruitment	The CovIdentify study launched on April 2, 2020 (Duke University Institutional Review Board #2020-0412). Eligibility criteria included age over 18 years and internet access. Social networks and social media advertising were used to recruit participants. By May 25, 2021, a total of 7,348 participants were recruited and e-consented through the Research Electronic Data Capture (REDCap) system. Of those consented, 6,765 participants enrolled in the study (Supplementary Table 1) by completing an enrollment survey. Soon after CovIdentify was launched, exploratory data analysis revealed major differences between CovIdentify demographics and the demographics of COVID-19 positive cases and deaths in the U.S., as well as overall U.S. demographics based on the 2020 U.S. Census. We sought to mitigate the imbalance throughout the duration of the study by providing wearable devices to underrepresented populations.<br><br>The recruitment details for the MyPHD study is provided in details in Mishra et al. (Nature BME 2020) and Alavi et al. (Nature Medicine 2021).
Ethics oversight	Duke University Institutional Review Board #2020-0412, Stanford University Institutional Review Board (IRB 55577, 57022, 23602), Stanford University Data Risk Assessment (#665)

Note that full information on the approval of the study protocol must also be provided in the manuscript.
